# Awareness and willingness to use HIV pre-exposure prophylaxis among men who have sex with men in low- and middle-income countries: a systematic review and meta-analysis

**DOI:** 10.7448/IAS.20.1.21580

**Published:** 2017-06-26

**Authors:** Siyan Yi, Sovannary Tuot, Grace W Mwai, Chanrith Ngin, Kolab Chhim, Khoundyla Pal, Ewemade Igbinedion, Paula Holland, Sok Chamreun Choub, Gitau Mburu

**Affiliations:** ^a^ KHANA Center for Population Health Research, Phnom Penh, Cambodia; ^b^ Center for Global Health Research, Touro University, Vallejo, California, USA; ^c^ Brighton and Sussex University Hospitals, Brighton, UK; ^d^ Division of Health Sciences, University of Warwick, Coventry, UK; ^e^ Division of Health Research, Lancaster University, Lancaster, UK

**Keywords:** HIV, pre-exposure prophylaxis (PrEP), men who have sex with men (MSM), low and middle income countries, systematic review

## Abstract

**Introduction**: To facilitate provision of pre-exposure prophylaxis (PrEP) in low- and middle-income countries (LMIC), a better understanding of potential demand and user preferences is required. This review assessed awareness and willingness to use oral PrEP among men who have sex with men (MSM) in LMIC.

**Methods**: Electronic literature search of Cochrane library, Embase, PubMed, PsychINFO, CINHAL, Web of Science, and Google Scholar was conducted between July and September 2016. Reference lists of relevant studies were searched, and three authors contacted for additional data. Non-peer reviewed publications were excluded. Studies were screened for inclusion, and relevant data abstracted, assessed for bias, and synthesized.

**Results**: In total, 2186 records were identified, of which 23 studies involving 14,040 MSM from LMIC were included. The proportion of MSM who were aware of PrEP was low at 29.7% (95% CI: 16.9–44.3). However, the proportion willing to use PrEP was higher, at 64.4% (95% CI: 53.3–74.8). Proportions of MSM aware of PrEP was <50% in 11 studies and 50–70% in 3 studies, while willingness to use PrEP was <50% in 6 studies, 50–70% in 9 studies, and over 80% in 5 studies. Several factors affected willingness to use PrEP. At the individual domain, poor knowledge of PrEP, doubts about its effectiveness, fear of side effects, low perception of HIV risk, and the need to adhere or take medicines frequently reduced willingness to use PrEP, while PrEP education and motivation to maintain good health were facilitators of potential use. Demographic factors (education, age, and migration) influenced both awareness and willingness to use PrEP, but their effects were not consistent across studies. At the social domain, anticipated stigma from peers, partners, and family members related to sexual orientation, PrEP, or HIV status were barriers to potential use of PrEP, while partner, peer, and family support were facilitators of potential use. At the structural domain, concerns regarding attitudes of healthcare providers, quality assurance, data protection, and cost were determinants of potential use.

**Conclusions**: This review found that despite low levels of awareness of PrEP, MSM in LMIC are willing to use it if they are supported appropriately to deal with a range of individual, social, and structural barriers.

## Introduction

Human immunodeficiency virus (HIV) is a leading cause of the global burden of disease [1] and mortality [2]. Currently, 36.7 million people are living with HIV globally, and on average, 1.1 million people die from it annually [3]. Although significant progress has been made in increasing access to antiretroviral therapy [2,3], world-wide incidence of HIV has remained above two million cases annually over the last decade [3], suggesting that additional HIV prevention interventions are required.

HIV pre-exposure prophylaxis (PrEP) is the provision of antiretroviral (ARV) drugs to HIV-uninfected people at high risk before potential exposure, to block the acquisition of HIV [4,5]. In randomized clinical trials (RCTs) conducted in high-, middle-, and low-income countries, PrEP reduced the risk of HIV acquisition by 44% among men who have sex with men (MSM) and transgender (TG) populations [6], 48% among people who inject drugs [7], and 67% among heterosexual serodiscordant couples [8]. In recent RCTs conducted in the UK and France, PrEP reduced the risk of HIV acquisition by 86% among MSM [9,10]. In all these studies and their open-label extensions, significantly higher levels of protection from HIV were experienced by participants who were adherent to PrEP [11].

The World Health Organization (WHO) recommended the use of daily oral PrEP to reduce HIV acquisition by HIV-negative partners within serodiscordant heterosexual couples in 2012 [4]. In 2015, WHO expanded the recommendation to include MSM and people who inject drugs [5]. WHO recommends PrEP to be used as part of a package of combination prevention interventions [5] that includes HIV testing, condom use, as well as screening and treatment of sexually transmitted infections (STIs) [4].

Following these recommendations, studies [12,13] and reviews [14,15] exploring awareness, willingness to use, and acceptability of PrEP among MSM have started to emerge over the last few years. However, these recent reviews have been broad in scope, and have included data from high-income countries. In his review, Holt [14] focused on acceptability of PrEP and use of ARV treatment as prevention among MSM in the Americas and Asia pacific, while Young and McDaid [15] focused on global acceptability of PrEP among all populations. While both reviews found that PrEP and treatment as prevention are reasonably acceptable, the over-representation of studies from high-income countries in both reviews, as well as the mixed populations in Young and McDaid’s review [15], limits the extent to which the findings may be applicable to MSM in low- and middle-income counties, where implementation of PrEP has been relatively limited compared to high-income countries [15,16].

In addition, Young and McDaid’s review [15] highlighted an urgent need to better understand motivations for willingness to use PrEP beyond clinical trials. Such information will inform practical ways in which MSM and other potential users can be best supported to access and utilize PrEP, particularly in low-and middle-income countries where experience with PrEP is limited. To respond to these information needs, we sought to examine the awareness of and factors associated with willingness to use oral PrEP among MSM in low-and middle-income countries.

## Methods

This review was conducted in accordance with the preferred reporting items for systematic review and meta-analyses [17] and protocols [18]. The protocol was registered in PROSPERO (ID: CRD42016043994) and the PRISMA checklist is appended as **supplemental file 1**.

## Search strategy

Between July and September 2016, a search was conducted in Cochrane library, Embase, PubMed, PsychINFO, CINHAL, Web of Science, and Google Scholar to identify relevant peer-reviewed articles related to awareness and willingness to use PrEP among MSM in low-and middle-income countries. Non-peer reviewed literature was not included. The search string utilized a combination of relevant keywords and was adapted for use with each database, using Boolean operators, truncations, proximity operators, and Medical Subject Heading (MeSH), as appropriate. An illustrative search used in PubMed is shown in **supplemental file 2**. To identify additional relevant citations, reference lists of included papers as well as “cited by” and “related citation” tools in Google Scholar and PubMed, respectively, were used. Three authors of ongoing studies and abstracts were contacted for information regarding additional data and peer-reviewed publications. No other limits were applied.

## Inclusion and exclusion criteria

### Study design

All study designs, including quantitative and qualitative studies, were considered eligible. Qualitative evidence regarding participants’ perspectives was included to provide a context for quantitative findings [19]. Non-original research, secondary reports, commentaries, editorials, and reviews were excluded.

### Domain

Studies were included if they were related to oral PrEP for HIV prevention. Studies that did not report findings related to PrEP were excluded.

### Population

Studies were included if they reported data generated from HIV-negative MSM in LMIC, regardless of age. Studies that reported data from MSM together with other populations such as TG or sex workers were included, but only data related to MSM were considered and abstracted.

### Intervention

**Figure 1.**
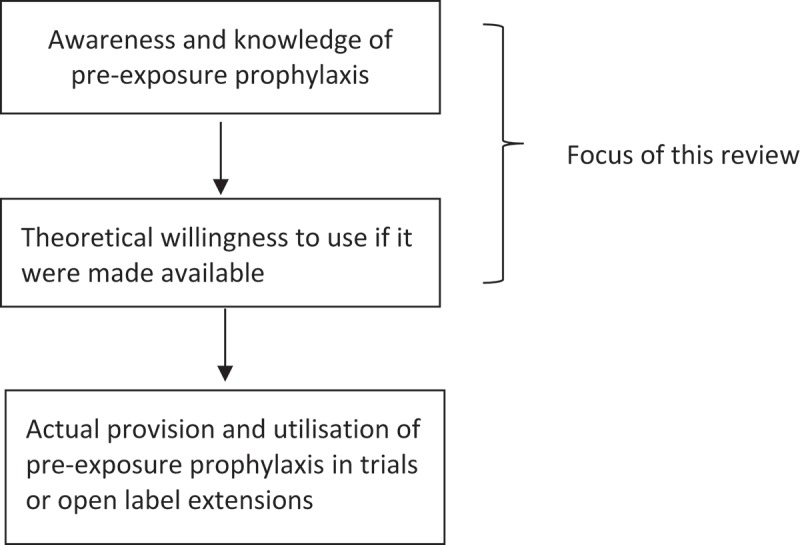
Scope of this review.

The review included studies that reported awareness or willingness to use PrEP. Studies that involved actual provision of PrEP and reported acceptability, such as clinical trials and open-label extensions of trials, were not the focus of this study and were excluded (Figure 1).

### Comparator

As this was a descriptive review, studies were included regardless of whether they reported outcomes from a control or counterfactual arm.

### Outcomes

Relevant patient-related primary outcomes included awareness of and willingness to use oral PrEP as shown in Table 1 below. The review elaborated on factors affecting the potential willingness to use PrEP where these were provided in the included studies, and mapped these factors across individual, social, and structural contexts of the socio-ecological model. The socio-ecological model is based on the assumption that individual health is determined by factors that are located within an individual, as well as those in their environment [20]. The model has been employed by other scholars to map the location of factors that affect health service utilization [21,22].

**Table 1.** Primary and secondary outcomes of this review

Outcome level	Definition
Awareness of PrEP	Proportion of MSM participants who reported knowing about PrEP
Willingness to use PrEP	Proportion of MSM participants who reported being willing to use PrEP if it was available
Factors affecting willingness to use PrEP	Individual, social, or structural factors that may determine the potential future use of PrEP

## Study selection, and data abstraction and management

Using End-Note software version 7 (http://endnote.com/), all citations were imported and duplicates removed. Three review team members independently screened references in two stages. In the first stage, titles and abstracts were screened to exclude ineligible studies based on relevance. In the second stage, full-text versions of selected papers were assessed independently by three reviewers to ensure that inclusion criteria were met. At each stage, selected papers were compared between the three reviewers for concordance. Screening and selection of studies were facilitated by the creation of appropriately labelled sub-folders in EndNote. In the event of uncertainty or disagreement, the three reviewers conferred and discussed with each other to reach a consensus. Data were abstracted into a standardized form with the following fields: authors, year of publication, country of study, design, settings, study populations, outcomes and limitations. To aid conceptual understanding of qualitative findings, typical participant quotes relating to awareness or willingness to use PrEP were also abstracted. A translator was utilized to translate three Chinese abstracts and the corresponding full papers which were subsequently included in the review.

## Data analyses

All available data were pooled and synthesized using a combination of a meta-analysis and narrative synthesis approach. The latter uses descriptive words and texts to summarize and explain results from a review [23]. For quantitative studies, proportions of participants who were aware or willing to use PrEP were abstracted and reported. A synthesis of factors determining willingness to use PrEP was performed, and these were classified as being individual, social, or structural in nature. Qualitative data were drawn on to provide context for the quantitative findings as recommended [19,24], by identifying participant perspectives about factors that may affect awareness and willingness to use PrEP among MSM. Using thematic analysis [25–27], relevant quotes were abstracted, sorted, compared, and categorized to construct a set of emerging descriptive themes. Themes were then used to populate a conceptual framework [28] of willingness to use PrEP at the individual, social, and structural domains. In keeping with the review protocol, meta-analyses were performed on the primary quantitative outcomes using a random effects model for pooling proportions [29], but pooled results from fixed effects models were displayed in graphical outputs to aid comparison and discussion. Subgroup analyses were not performed.

## Bias assessment

Three reviewers evaluated the risk of different types of biases, including selection bias, attrition bias, and information and reporting bias using methodology suggested in the Cochrane Collaboration’s tool for assessing risk of bias [30]. Studies were included regardless of risk of bias, but the impact of their inclusion on the robustness of findings and conclusions was discussed.

## Results

### Study selection

**Figure 2.**
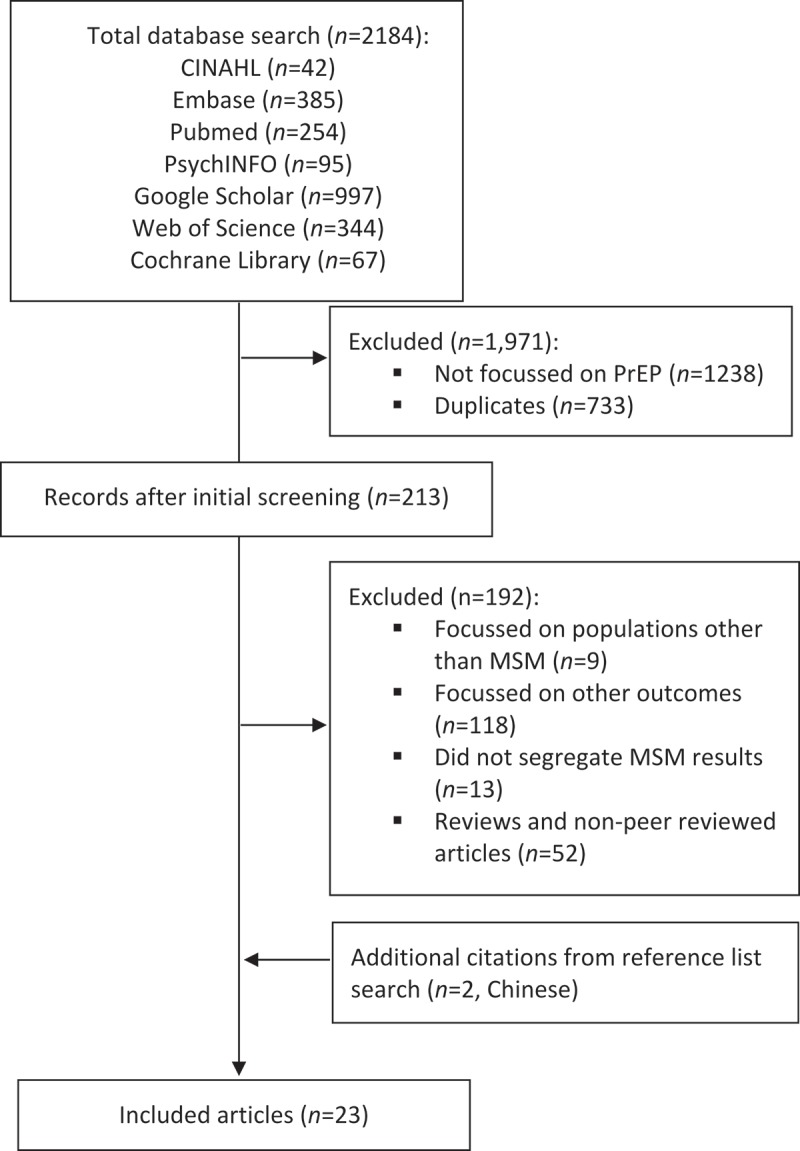
Study selection.

This review involved a total of 2186 records. The initial screening excluded duplicates (*n *= 733) and studies that did not specifically focus on PrEP (*n *= 1238), leaving a total of 213 citations. Subsequently, 192 citations were excluded after screening the abstracts and full papers. A few papers focusing on other populations (e.g. heterosexual couples, sex workers, and TG populations) had been identified through the search and were excluded (*n *= 9). Additional exclusions were due to a variety of reasons, including poor relevance of outcomes (e.g. cost effectiveness; *n *= 118), failure to segregate results by population (*n *= 13), and for being reviews (*n *= 7) or non-peer reviewed articles (*n *= 45). In total, 2161 records from the initial search were rejected for failing to meet the inclusion criteria. An additional two Chinese citations were identified from reference lists of included papers. Three authors were contacted to provide full papers or additional data to that reported in their abstracts, but these papers were not additional as they had been identified in the original search. In accordance with PRISMA guidelines for a systematic review [17], a flow diagram illustrating the literature search, article selection, and final included studies is shown below (Figure 2).

### Methods and study designs of included studies

We included 23 studies published between 2011 and 2016, and involving 15,014 MSM, of whom 14,040 were from LMIC. Of these, three were published in Chinese, and the rest were in English. Of the included studies, 19 were quantitative, two were qualitative, and two were mixed methods. These 23 studies related to 22 distinct populations. Two Malaysian studies conducted by Lim et al. [31] and Bourne et al. [32] were linked in that participants of the quantitative study were invited to participate in follow-on qualitative interviews. All of the quantitative studies were cross-sectional surveys. The study by Wheelock et al. [33] was a replication of that by Eisingerich et al. [34], but in a different country. The following tables present the key characteristics of the quantitative (Table 2) mixed-methods (Table 3) and qualitative studies (Table 4) included in the review.

**Table 2.** Characteristics and findings of quantitative studies included in the systematic review

Author, year	Country and setting	Design	Sample size	Participants’ characteristics	Awareness	Willingness to use	Factors associated with willingness to use
Ayala et al., 2013	145 countries- Africa, Asia, Europe and Latin America.	Online survey.	2774 MSM.^α^	Age range was 12–90 years.	69.8% of the respondents were aware.^α^	80.8% were willing to use PrEP. ^α^	PrEP stigma (*β*: −0.51; 95% CI= −0.55 to −0.48, *p*<0.001), outness (*β*: −0.15; 95% CI= −0.18 to −0.12, *p*<0.001), and knowledge about PrEP (*β*: −0.14; 95% CI= −0.18 to −0.10, *p*<0.001) were negatively correlated with acceptability of PrEP. Acceptability of PrEP was positively correlated with having experienced service provider stigma (*β*: 0.12; 95% CI=0.02–0.23, *p*=0.021). Respondents in high-income countries reported lower acceptability of PrEP than those from LMIC.
Ding et al.,2016	Shanghai, China.	Survey.	1,033 MSM.	76% self- identified as gay and 2.5% were already using PrEP.	Not reported.	19.1% willing to use PrEP.	Willingness to use PrEP was associated with older age (≥ 45 years (Adjusted Odds Ratio (AOR):2.18; 95% (Confidence Interval (CI)=1.13–4.23, *p*=0.006), immigration to Shanghai (21.5% among immigrants vs 15.9% among local residents; AOR: 1.69; 95% CI=1.16–2.45, *p*=0.026), two or more male sex partners in the past 6 months (AOR:1.53; 95% CI=1.07–2.17, *p*=0.02). Condom use at last anal sex with man were significantly less willing to use PrEP (AOR:0.68; 95% CI=0.47–0.97, *p*=0.034). Education, occupation, gay sexual identity, and marital status were not associated with willingness to use it.
Draper et al., 2016	Yangon and Mandalay in Myanmar.	Survey.	434 GMT^β^	Not reported.	5% aware.	62% were willing to use PrEP among 434 HIV undiagnosed GMT.	Willingness to use PrEP was associated with reporting never/occasional use of condoms compared to always/mostly used, with casual partners (adjusted odds ratio (AOR: 2.02; 95% CI=1.00–4.10), residence Mandalay (AOR:1.79; 95% CI=1.05-3.03), perceiving as likely to become HIV positive (AOR:1.82; 95% CI=1.10–3.02), having had more than one regular partner (AOR=2.94; 95% CI=1.41–6.14) or no regular partners (AOR:2.05; 95% CI=1.10–3.67) or more than five casual partners (AOR:2.05; 95% CI=1.06–3.99) or no casual partners (AOR:2.25; 95% CI=1.23–4.11) in the past three months. Those reporting concerns about PrEP side-effects due to long-term use were less likely to be willing to use it (AOR:0.35; 95% CI=0.21–0.59).
Eisingerich et al.,2012	Peru, India, South Africa.	Survey	383 MSM^γ^	Mean age of MSM not reported; 39% were aged 16-24, and 6% were aged ≥41 years.	Not reported	69% reported ‘yes, definitely’ and 25% ‘yes, probably’ across India, Peru and South Africa**^β^**.	42–69% reported that PrEP would give them “a lot of hope”. 3–8% reported that PrEP would be “very embarrassing” to take. Indian and Peruvian MSM preferred bimonthly injection in the buttocks while South African MSM preferred daily pill to arm injection. Of those willing to use PrEP, 32–72% were willing to use PrEP despite side effects; 39–88% were willing to use it despite having to pay, 32–85% were willing to use it even if having to use condoms, and 55–88% were willing to use it with regular HIV testing.
He et al., 2014	China.	Survey.	1323 MSM^δ^	Mean age=28 years.	Overall, 31.4% had heard of PrEP.	Not reported.	Factors affecting use were not reported; however, the study reported that information regarding PrEP should be promoted through media to make sure MSM in China can get the information quickly and easily.
Hoagland et al., 2016	Brazil.	Cross-sectional study.	1131 MSM^ε^	Median age=29 years 46.8% were HIV positive.	61.3% were aware.	82.1% were willing to use PrEP.	Willingness to use PrEP was higher among those aware of PrEP compared to those unaware of it (85.4% vs 76.9% ;p<0.001), among those with more years of schooling (78.1% among those with <12 years vs 84.5% among those with ≥12 years of schooling; *p*=0.006), and those with a recent STD diagnosis in last 12 months compared to those without (68.8% vs 60.2%; p=0.02). Willingness to use PrEP was not associated with age. Compared with those aged 18–24 years, willingness to use PrEP increased marginally among 25–35year olds (81.02%, vs 81.7%; *p*=0.85) and among those aged ≥36years (81.02% vs 84.1%; *p*=0.35). Willingness to use was not associated with male gender compared to transgender (81.8% vs 89.3%; *p*=0.16), a negative compared to a positive HIV test result (62.7% vs 46.8%; p=0.99), or failure to perform a test (62.7% vs 65.4%; *p*=0.40). 75.8 % reported they would use PrEP even if they had to pay for it.
Jackson et al., 2012	Guangxi, Sichuan and Chongqing, China.	Survey.	570 MSM	Mean age=27.6 years; age range=18–62 years, and 76.8% were urban dwellers.	Not reported.	63% had high willingness to use PrEP, while 22.8% had lower willingness to use it.	Willingness to use PrEP was associated with urban compared than rural residence, higher education attainment (2.2% among primary school, vs 10.3% among middle school, vs 38.2% high school vs 49.3% among those with undergraduate or higher education; p<0.001), lower monthly personal income (37.9% among those earning 1,000 Yuan or less vs 2.8% among those earning 5,000 Yuan or more. Occupational status and previous experience of STI were not associated with willingness to use PrEP. Stigma of PrEP was a potential barrier, while perceived benefits of PrEP was a facilitators of potential use.
Ko et al., 2016	Taiwan.	Online survey.	1151 MSM	Mean age=25.9 years, age range=18–53 years, most were from the north (48.5%), had professional qualification (61.2%) and were employed (57.0%).	Not reported	56% were willing to use PrEP.	Of those willing to use PrEP, 70% were willing to take pills before and after sex, 61% were willing to take PrEP to prevent getting HIV, 43.7% were willing to take a pill daily, 44.4% were willing to take PrEP even if it was not 100% effective, and only 23% were willing to self-pay Taiwan $ 340 for PrEP. Willingness to use PrEP increased with tertiary compared with secondary education (30.7% vs 2.2%; p<0.05), and among those with professional qualification (54.8% vs 12.2%; p<0.05), and a past history of receiving HIV non-occupational PEP (5.9% vs 3.2%; p value <0.01). There was no difference in age or employment between participants who were willing to use PrEP and those who were not.
Lim et al., 2016	Kualar Lumpur, Malaysia.	Survey (online).	990 MSM^ϕ^	80.4% self-identified as homosexual and 16.6% as bisexual. Age range=16–68. Overall 19.6% were aged <25 years. In addition, 87.2% had post-secondary education and 85.2% were in part-time or full time employment.	44% were aware of PrEP.	39% were willing to use PrEP.	Recent STI diagnosis in the past 12 months was associated with high likelihood to use compared to those with no such diagnosis (43.3% vs 36.1%; *p*=0.003). Malay participants more likely to use PrEP (48.6%) compared to Chinese (32.7%), Indian (32.8%) and mixed and other races (36.7%; p<0.001). Willingness to use PrEP was not associated with age, residence in Kuala Lumpur, education, employment status, or income. A third (35.6%) were willing to pay for PrEP. However, of these the majority (88.3%) were not willing to spend over 200 RM (USD 50) on PrEP per month. Of the 603 participants who reported not willing to use PrEP, the reasons offered were side effects (18.6%), fear that PrEP won’t work (9.8%), worry about forgetting to take medication (8.3%), or what other people might think of them (5.8%), failure to afford PrEP (8.8%), or the fact that they always use a condom and therefore would not need PrEP (11.4%).
Oldenburg et al., 2016	Ho Chi Minh city, Vietnam.	Survey.	300 MSM^γ^	93.7% were HIV negative, and 27% were aged 15–19 years.	Not reported.	95.4% were willing to use PrEP daily.^η^	Overall, 56.7% willing to take PrEP given side effects, and 27.7% preferred a PrEP lubricant to a pill. Previous contact with Peer Health Educators was associated with higher willingness to use (AOR: 2.28; 95% CI=1.25–4.14, *p*<0.05).
Peinado et al., 2013	Lima, Iquitos and Pucallpa, Peru.	Survey (secondary analysis).	532 MSM and TG^ι^	Median age=28 years; range 16–68 years.	Not reported.	96.2% were willing to use oral PrEP while 91.7% were willing to use rectal PrEP	After adjustment for age, city, and education, only being receptive most of the time (AOR: 9.1; 95% CI=1.8–46.5, *p*=0.01) and exclusively receptive (AOR:7.5; 95% CI=1.6-53.2, *p*=0.01) during anal intercourse, compared to being versatile, were independently associated with acceptability to use oral PrEP.
Sineath et al., 2013	Thailand.	Survey (online)	404 MSM.^φ^	Mean age was=25 years.	7% were aware of PrEP.	36% were willing to use after PrEP was described.	Of those willing to use PrEP 65% indicated they would be willing to pay for it. Overall, 34% “didn’t want to have to take medication every day” and 28% “didn’t want to go see the doctor every three months”. In addition, 35% believed condoms were more effective than PrEP.
Wei et al., 2011	Guangxi, China.	Survey (face to face).	650 MSM.	Mean age=28 years	19.7% had heard about PrEP.	91.9% were willing to use PrEP if free and safe.	Side effects and efficacy of PrEP were reported as influencing willingness to use.
Wheelock et al., 2013	Bangkok and Chiang Mai, Thailand.	Survey.	260 MSM.	4% and 54% were 16–18 and 19–24 years, respectively. Eligible participants were at least 16 years. 94% had post-secondary education.	Not reported.	39.2% reported they would ‘definitely’ and 49.2% would ‘probably’ use PrEP.	Of those willing to use PrEP, 58.8% were ‘definitely’ while 35% were ‘probably’ willing to use PrEP despite having to pay 500 Baht a month for it. 2.7% reported that taking PrEP would be ‘very embarrassing’ and 5.8% reported that it would be ‘fairly embarrassing’. Daily pill was the preferred route of administration followed by a monthly injection in the arm. After learning of potential mild side effects, 24.6% were ‘definitely’ and 56.5% ‘probably’ willing to use PrEP.
Xia et al., 2016	Wuhan and Shanghai, China.	Survey.	487 MSM	Mean age=28; range 18–62; years. 31.7% were aged 18–24 and 53.5% were aged 25–34 years. 81.1% self-identified as gay, and 16.2% as bisexual. 73% were educated to college level, 61.4% were employed, 51.1% earned between 2001–5000 RMB and 7% had been diagnosed with an STI in the last year.	19.1% aware.	71.3% willing to use.	Willingness to use PrEP was associated with marital status: 84.4% of those married/cohabiting were willing to use PrEP versus 67.5% of unmarried/divorced or widowed (*p*=0.001). Bisexual (77.2%) were more likely to use it compared to gay participants (71.1%) or /other/unsure (38.5%; *p*=0.017). Willingness to use was associated with taking an STI test in the last 12 months compared to those that didn’t (76% vs. 63.4%; *p*=0.007) but was inversely associated with being diagnosed with an STI in last 12 months compared to those not diagnosed, though not significant (67.6% vs 72.6%; *p*=0.065). Men using the internet were more likely to report willingness to use PrEP compared to those who heard about PrEP face-to-face (75.2% vs 66.4%; *p*<0.05). Willingness to use was not associated with age or duration of residency in the city.
Xue et al. 2015	China.	Survey (online).	760 MSM**^κ^**	77.2% self-identified as homosexual and 20% remainder as bisexual	72.8% aware of, or fully understood PrEP.	32.1% would possibly use PrEP.	61% (305/500) would possibly take PrEP orally daily. Factors that were identified by participants as preventing willingness to use PrEP were: side effects (60.8%), low self-risk assessment (54.2%), privacy and confidentiality (41.6%), the perception that PrEP is not 100% effective (38.3%), cost (28.7%), inconvenience of taking daily medication (68.7%), and reporting that risk behaviors were not happening daily (59%).
Yang et al., 2012	Chiang Mai, Thailand.	Survey.	131 MSM**^λ^**	Mean age=23.7; range 18–49 years.^μ^ 13% self-identified as heterosexual, 16% as bisexual and 71% as gay.	66% aware of PrEP.	41% willing to use PrEP.^ν^	Willingness to use PrEP among MSM was associated with having zero regular partners in the preceding 6 months vs. one or more partners (OR: 2.25; 95% CI=1.09–5.11, *p*=0.04); regularly planned sex vs. unplanned sex (OR:2.83; 95% CI=1.12–7.12, *p*=0.01); infrequent sex (once per month or less) vs. two or more sexual encounters per month (OR:2.36; *p*=0.02); a lifetime history of STIs vs. no history of STIs (OR 3.78, 95% CI=1.42–10.47, *p*<0.01); age 25 years or older vs. age less than 25 years (OR:2.30; 95% CI=1.10–4.79, *p*=0.02); and being “very confident” in the ability to take daily, oral medicines for 1 year vs. not being “very confident” (OR:2.63; 95%CI=1.12–6.24, *p*=0.01). In contrast, willingness to use was not associated with a lifetime history of HIV testing vs. no history of HIV testing (OR:1.95; 95% CI=0.89–4.29, *p*=0.07) or receptive anal sex positioning vs. insertive or versatile positioning (OR:0.47; 95% CI=0.17–1.19, *p*=0.08)
Zhang et al., 2013	Chongqing, Guangxi, and Sichuan, China.	Survey.	1402 MSM^ο^	Age range=18–74 years. 18–24 years comprised 41.5% of the sample. Majority (75.1%) resided in urban areas. 70% self-identified as homosexual and 21% as bisexual.	22% were aware of PrEP	64% were willing to use PrEP if safe and effective.	Proportion willing to use PrEP increased to 71% if it were to be made free, and to 77% if it were free and had been used by people known to participants. However, only 30% and 37% were willing to use it if it had to be taken once daily or a weekly respectively. Willingness to use PrEP was associated with lower education up to middle school compared to those with college education and above (68.4% vs 59.5%; p=<0.001), married marital status compared to never married (69.7% vs 62.4%; *p*=0.035); moderate (1000-3000) monthly income (compared to lower earnings of <1000; (*p*=0.013) but not compared to high monthly income of >3000 (*p*=0.109); and STI history compared to those without STI history (71.9% vs 62.6%; *p*=0.027). Participants who did not or rarely found sexual partners on the internet were more likely to be willing to use PrEP compared with higher risk participants, who often or sometimes found sexual partners on the internet. Willingness to use was not associated with age or residence or sexual identity.
Zhou et al., 2012	Beijing, China.	Survey.	152 MSM^π^	Age range=18–61 years. 84.9% self-identified as homosexual and 15.1% as bisexual.	11.2% aware of PrEP.	67.8% were willing to ‘definitely’ or ‘probably’ take PrEP if available.	Willingness to use PrEP was associated with young age <30 years versus ≥30 years (68.8% vs. 83.9%; p=0.04). Willingness to use PrEP was not associated with years of education (80% among those with <12 vs 68.1% among those with >12 year of education; *p*=0.09), marital status (single/divorced/ widowed versus married/cohabiting [73.1% vs 77.1%; p=0.60), local Beijing residence versus non-Beijing residence (63.3% vs 77.1%; p=0.13), lower monthly income (RMB) <2000 versus >2000 (77.1% vs 72%; *p*=0.47; bisexual orientation versus homosexual (73.7% vs 74.7%; *p*=0.89], or previous diagnosis of STD in the past 6 months versus no such diagnosis (88.9% vs 72.4%; *p*=0.15. Participants expressed worry about side effects (63.8%), lack of prevention efficacy in PrEP (44.1%), diet and sleep disruption by PrEP (44.7%), development of resistance from PrEP (21.7%), being treated as an AIDS patient by people (20.1%), being refused sex by male partners after using ARV drugs (14.5%) or not being able to afford ARV drugs (26.3%).

^α^ Total participants in this study were 3748, and were from 145 countries globally, including Asia (26%), Caribbean (2%), Eastern Europe and Central Asia (17%), Latin America 567 (15%), Middle East and North Africa (2%), Oceania (6%), sub-Saharan Africa (5%), and western and Northern Europe and North America (26%). Awareness and willingness to use data reported here relate to 2774 LMIC participants only; global awareness and willingness to use PrEP were 72% and 82%, respectively.

^β^ Participants included gay men, other men who have sex with men and transgender participants (GMT). Among 434 of 520 were HIV undiagnosed GMT and 17% (n = 86) were HIV positive.

^χ^ The overall sample was 1790, which included MSM, FSWs, IDUs in Peru, Ukraine, India, Kenya, Botswana, Uganda, and South Africa. However, MSM (n = 383) were sampled in Peru, India, South Africa.

^δ^ 1407 MSM were approached, but only 1323 questionnaires completed and analyzed.

^ε^ The overall sample was 1187 of whom 95.3% were male and 4.7% were transgender participants.

^ϕ^ A total of 2,644 participants were screened from whom the 990 were included.

^γ^ This was an exclusive sample of MSM who were also sex workers.

^η^ Among the 93.7% (n = 281) HIV-negative individuals in the study.

^ι^ Proportion of MSM vs. TG was not stated.

^φ^ 470 MSM took part in the survey but 404 completed the survey and were included in the analysis.

^κ^ A total of 887 MSM started to fill questionnaire, but only 760 qualified questionnaires were analyzed.

^λ^ 326 individuals completed the screening questionnaire out of which 238 MSM and TG were eligible and completed the survey (131 MSM and 107 TG)

^μ^ Mean age reported here is that of MSM participants only.

^ν^ Willingness reported here is among MSM participants, and excludes transgender participants.

^ο^ 1407 MSM were recruited, but 1402 completed the questionnaires and were analyzed in the study.

^π^ 159 MSM were enrolled, but only 152 used for analysis as 7 were deleted for not having sex with men in the past 6 months.

FSW: female sex worker; GMT: gay, men who have sex with men and transgender; HIV: human immunodeficiency virus; MSM: men who have sex with men; IDU: injecting drug user; PEP: post-exposure prophylaxis; Taiwan $: Taiwan dollar; PrEP: pre-exposure prophylaxis; RMB: Ren Min Bi (currency of People’s Republic of China); STI: sexually transmitted infection; STD: sexually transmitted disease; TG: transgender.

**Table 3.** Characteristics and findings of mixed-methods studies included in the systematic review

Author, year	Country and setting	Design	Sample size	Participants’ characteristics	Awareness	Willingness to use	Factors associated with willingness to use
Galea et al. [50]	Lima, Peru	Mixed methods (FGDs and conjoint analysis)	17 MSM.^α^	Mean age for MSM = 33 years	Little or no awareness of PrEP	Participants were supportive of using PrEP, but had various concerns	High out-of-pocket cost, partial efficacy, and fear of side effects, stigma and discrimination were associated with PrEP use, while mistrust of health-care professionals and a belief that PrEP would result in a decrease in condom use were concerns for MSM. Participants preferred PrEP provided at healthcare centres as opposed to pharmacies, due to cost.
Karuga et al. [51]	Kisumu, Kenya	Mixed methods.	80 MSM	Median age = 24.9 years. 68.8% were HIV negative, 11.8% were sex workers, and 49.1% were exclusively homosexual	Precise proportion not reported, but in-depth knowledge of PrEP was noted to be low	83.3% were willing to use PrEP if made available.^β^	Willingness to use PrEP was associated with sexual orientation, being higher among bisexual compared to homosexual (96.2% vs. 74.1%; p = 0.025). Willingness to use PrEP was not associated with age (p = 0.616), or university compared to secondary education (81.2% vs. 89.5%; p = 0.470), or marital status (p = 0.157). Stigma, general dislike of taking medicines, uncertainty over PrEP effectiveness, cost, and a lack of information were reported as influencing willingness to use PrEP from qualitative interviews.

^α^ The overall sample was 45 including 15 FSW, 13 TG, and 17 MSM. Figures for each country were reported separately but have been averaged here.

^β^ Willingness reported is among the 55 HIV-negative MSM.

FSW: female sex worker; FGDs: focus group discussions; MSM: men who have sex with men; PrEP: pre-exposure prophylaxis; TG: transgender.

**Table 4.** Characteristics and findings of qualitative studies included in the systematic review

Author, year	Country and setting	Design	Sample size	Participants’ characteristics	Awareness	Willingness to use	Factors associated with willingness to use
Bourne et al. [32]	Kuala Lumpur, Malaysia.	Qualitative	18 MSM.	Not reported, but eligibility was ≥18 years of age.	Not reported.	Most MSM were willing to use PrEP but concerned about a range of barriers.	Participants would consider PrEP in future if they had higher number of concurrent sexual partners and if PrEP were free or cost a maximum of RM 50–200 (USD 12–49) per user per month. Barriers included potential side effects, anticipated lack of discipline to take PrEP daily, confidentiality and data protection concerns, fear of stigma and being perceived as having riskier behaviours such as *barebacking* or ‘raw sex’ by non-PrEP using peers. The *physical* barrier of condoms was preferable to PrEP which can’t be seen or felt. Participants felt that PrEP may not be needed in monogamous relationships as use of condoms would be sufficient.
Chakrapani et al. [52]	Chennai and Mumbai, India.	Qualitative	61 MSM and 10 key informants^α^.	21.3% were bisexual and mean age = 26.1.	None of the participants were aware	55.7% would use PrEP if available	Stigma, shame, lack of trust, cost of PrEP, fake pills, and fear of side effects were barriers. Motivators included peace of mind when condoms break/slip, ‘additional protection’ in case condom breaks, desire to have safe sex with HIV-positive steady partners, and ability to take PrEP discreetly.

^α^Key informants (*n* = 10) included community leaders and healthcare providers. MSM: men who have sex with men; PrEP: pre-exposure prophylaxis; RM: Malaysia Ringgit; USD: USA dollar.

## Recruitment and data collection settings

Most studies were conducted in urban areas. Five studies used internet-based advertisement and recruitment, including Facebook or organizational websites [31,35,38], often in combination with mobile-based social dating applications (such as Grindr) [31] as well as TV and newspaper advertisements [38]. Several studies used face-to-face recruitment, exclusively or in combination with online methods. Five studies sampled participants from service provision sites such as community-based and youth-led non-governmental organizations [31,32,50,52]. Two studies utilized health facilities for recruitment and data collection, including community health clinics [53] and HIV voluntary counselling and testing sites [51]. Three studies utilized other venues frequented by MSM such as entertainment venues [33,47], gay community events [53], and beauty salons [50]. Other sites of recruitment included parks, volley-ball courts, and streets [50].

## Geographical location of included studies

Two studies conducted by Ayala et al. [35] and Eisingerich et al. [34] were multi-country in scope, while all the rest were conducted in a single country. The 21 single-country studies had MSM participants from Brazil, China, India, Kenya, Malaysia, Myanmar, Peru, Thailand, Uganda, Vietnam, and South Africa. China contributed to most studies (*n *= 8), followed by Peru (*n *= 3), and Thailand (*n *= 3). The study by Ayala et al. [35] was conducted in 145 countries, which included high-income countries as highlighted in Table 2. At the time of the review, almost all of the countries from which the participants in this review were based were classified by the World Bank as either middle- or upper-middle income, except Uganda which was a low-income country (http://data.worldbank.org/country).

## Description of participants in the included studies

Together, the included studies involved a total of 15,014 MSM. Of these 14,040 were from LMIC. (some few TG participants may be included where they were not separated from MSM in two papers). The profile of MSM included in the studies within the review included homosexual and bisexual MSM. In total, eight studies from Brazil [39], China [46,47,49], and Kenya [51] included participants who described themselves as bisexual. The proportion of participants that were bisexual was highest in the Kenyan study by Karuga et al. [51] at 50.1%. Several studies included MSM who were sex workers. In Karuga et al. [51], 11.8% of participants were sex workers, while, Oldenburg et al. [41] purposely recruited an exclusive sample of MSM sex workers.

Apart from MSM, several studies included other populations, although only data related to MSM were abstracted. For example, Galea et al. [50] focused on MSM, female sex workers, and TG populations in Peru. In their study, Eisingerich et al. [34] included intravenous drug users, serodiscordant couples, and young women in Ukraine, India, and other African countries. Studies by Peinado et al. [42], Yang et al. [47], and Hoagland et al. [39] included MSM and TG populations. These studies segregated the results by gender identity, and for the purpose of this review, only data related from MSM in these studies were considered. However, the 2016 study by Draper et al. [37] included MSM and TG participants, who were not fully segregated.

Given the relevance of PrEP for HIV prevention, most studies included HIV-negative status as an eligibility criterion. However, in the study by Karuga et al. [51], only 68% were HIV negative, because recruitment involved all MSM who were presenting for HIV testing, but only HIV-negative participants were asked about their willingness to use PrEP. Likewise, 46.8%, 18%, and 17% of initial participants in the studies by Hoagland et al. [39], Ayala et al. [35], and Draper et al. [37] were HIV positive, respectively. Similarly, in these studies, willingness data were generated from HIV-negative participants. The Thai study by Ding et al. [36] and the Malaysian study by Lim et al. [31] found that a small proportion of their participants were already using PrEP (2.5% and <1%, respectively). Finally, the profile of participants was also influenced by the recruitment strategies of the included studies, including eligibility criteria. While most studies recruited MSM older than 18 years, two studies had lower age eligibility criteria of 15 years [41] or 16 years [33].

## Bias assessment

Included studies had multiple sources of bias resulting from confounding, recruitment, non-response, social desirability, and attrition bias (**supplemental file 3**).

*Confounding*: All of the quantitative studies were cross-sectional in design and had high potential for bias and confounding. All studies reported hypothetical likelihood of using PrEP, which could change once PrEP is provided.

*Recruitment bias*: The risk of recruitment bias was significant in a number of studies, based on the methods and settings where recruitment took place. The study by Ayala et al. [35] included MSM participants from 145 countries globally, including Asia (26%), the Caribbean (2%), Eastern Europe and Central Asia (17%), Latin America (15%), Middle East and North Africa (2%), Oceania (6%), sub-Saharan Africa (5%), and western and Northern Europe and North America (26%). In this study [35], awareness and willingness to use data were segregated by geographic region. However, overall correlation statistics were not, and therefore participants from high-income settings may affect the reported correlation statistics. In the Indian study by Chakrapani et al. [52], participants were recruited exclusively through a community-based organization, an approach that may have excluded MSM who did not have contact with community-based HIV services. Sineath et al. [43] recruited a convenience sample of Thai MSM exclusively through online methods, which excluded those who did not have access to internet.

The studies by Lim et al. [31], Bourne et al. [32], and Jackson et al. [53] expanded their online-based recruitment to include recruitment through local non-governmental organizations, which may have facilitated inclusion of different profiles of MSM. A number of studies recruited specifically from sites which were thought to be frequented by high-risk MSM, including from entertainment venues in Thailand [33,47], gay community events in China [53], and beauty salons in Peru [50]. Although appropriate for identifying MSM who require PrEP, these strategies may exclude other MSM, for example those who are not living openly as MSM. A number of studies in Thailand [43], Malaysia [31,32], Kenya [51], and China [46], among others, started off by recruiting MSM and then screened them for eligibility based on age, HIV status, and availability and interest to participate in interviews, among other criteria, thereby limiting generalizability of findings to wider MSM populations. In addition, recruitment from rural areas across all studies was limited.

*Attrition and non-response bias*: The studies by Xue et al. [46], Sineath et al. [43], Zhang et al. [48], and He et al. [38] reported instances of non-responses or incompletely filled questionnaires, which introduced response bias and may limit generalizability.

*Social desirability bias*: Most of the measures reported in the included studies were self-reported, and therefore were prone to social desirability bias, especially self-reported sexual behaviours. Because of a social desire for positive self-presentation, willingness to use PrEP may have been over-reported by some participants in Peru who saw its use as responsible behaviour [50] while it could have been under-reported by other participants in Malaysia, India, Peru, and Thailand who thought it may be perceived it as a sign of promiscuity [32,33,50,52]. The potential for social desirability bias may have been mitigated by the use of anonymous online data collection methods in the studies by Sineath et al. [43], Lim et al. [31], and Ayala et al. [35], while being particularly accentuated in four studies by Bourne et al. [32], Chakrapani et al. [52], Karuga et al. [51], and Galea et al. [50], which involved face-to-face focus group discussions with peers. The two studies by Chakrapani et al. [52] and Karuga et al. [51] also involved in-depth interviews in addition to focus group discussions.

*Researcher bias*: The four studies that reported qualitative findings lacked clarity around reflexivity. They contained limited documentation of interview dynamics, emotions, interactions, or beliefs of the researchers as recommended [54], especially when dealing with sensitive topics [55]. Although absence of this information may have been occasioned by word count limitations, it limited our analysis of the researchers’ influence on the research conduct and reported findings, which is an essential element of evaluating qualitative research [25].

## Description of PrEP

The included studies used consistent definitions of PrEP and most explored oral PrEP except for the Peruvian study conducted by Peinado et al. [42], which explored both oral and rectal PrEP. However, findings in this study were segregated by route of administration. Most studies provided a definition of PrEP to participants and emphasized the need to adhere to medications as part of the definition.

In addition, four studies [33,34,41,50] explored participants’ preferences regarding the most desirable formulations of PrEP, such as injectable or oral or lubricant forms. However, these results did not affect the data reported in this review regarding willingness to use oral PrEP, as data were segregated.

Definitions and general information regarding PrEP were provided by researchers or data collectors who were facilitating interviews, focus group discussions [32,49,50,52], or face-to-face quantitative questionnaires [33,34,47]. In other studies, this information was provided online as part of the study [31,35]. In one Indian study, explanation of PrEP was facilitated by pictorial cards [52], while in several other studies [33,34,49,52], PrEP definitions were provided in both English and local languages to aid its understanding. At least three studies [31,32,50] made explicit attempts to differentiate PrEP from post-exposure prophylaxis (PEP) in their definitions of PrEP to participants, and in rare cases [52], data collectors were provided with fact sheets to respond consistently to participants’ queries.

## Nature of outcomes reported

Of the outcomes of interest to this review, the most reported outcome was willingness to use. All quantitative studies reported either proportions of participants who were aware of or willing to use PrEP, or both, with a number of them examining willingness to use PrEP in different situations related to efficacy, cost, knowledge of partners, condom use, and stigma [31,33,34,40,41,49]. Two qualitative [32,52] and two mixed-methods studies [50,51] explored perceptions and perceived barriers and facilitators of willingness to use PrEP, in relation to relationships, sex, PrEP information and education, cost, ways and venues to access PrEP, risk perception, ideal nature of PrEP, perceived effectiveness, side effects, and adherence issues.

Four studies [35,42,47,52] exploring willingness to use described this outcome as acceptability, even though they explored theoretical use of PrEP if it were made available. This information was abstracted and reported as willingness to use. Because this review excluded trials in which PrEP was being assessed, it excluded actual acceptability in which PrEP was provided as part of the study. In one study by Ding et al. [36], participants were assessed for willingness to use PrEP and were subsequently offered it. However, although the study noted the proportion of participants that eventually took up the PrEP (20.5% changed their minds), this review abstracted the initial willingness to use data, rather than the acceptance data. Nevertheless, as noted above, the study by Ding et al. [36] and that by Lim et al. [31] incidentally found that small proportions of participants (2.5% and <1%, respectively) were already accessing and using PrEP at the time of the study.

## Awareness of PrEP

**Figure 3.**
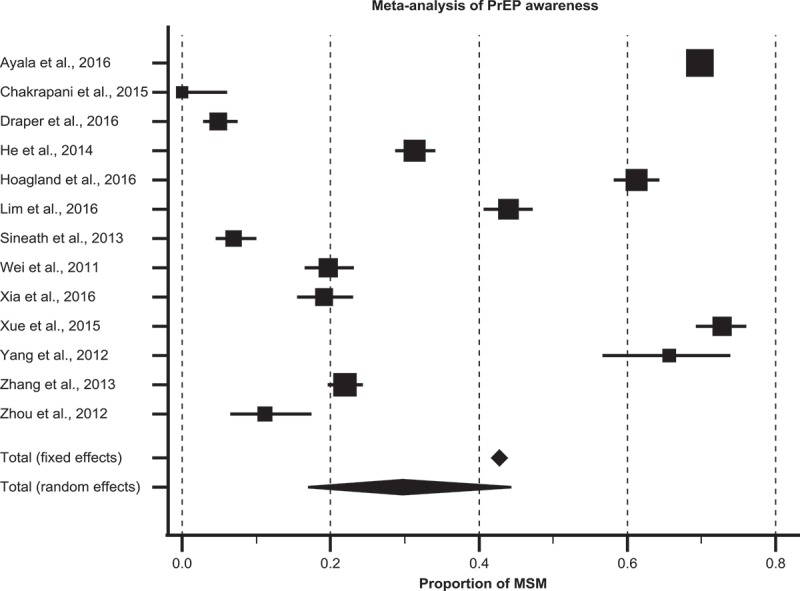
Pooled estimate of awareness of PrEP among MSM in low- and middle-income countries.

Of the 14 studies reporting levels of awareness, 13 provided quantitative proportions of participants who were aware of PrEP. All studies reporting awareness used a simple binary question asking participants whether they were aware (or had heard) of PrEP, which was a consistent measure of awareness across studies. Most studies that explored awareness found a lack of awareness of PrEP among participants. With the exception of four studies by Hoagland et al. in Brazil [39], Yang et al. in Thailand [47], Ayala et al. [35], and Xue et al. in China [46] that reported awareness of 61.3%, 66.0%, 69.8%, and 72.8% respectively, most studies reported much lower awareness of PrEP ranging from 5.0% in Myanmar [37], 7.0% in Thailand [43], 11.2% in China [49], 19.1%, 19.7%, 22%, and 31.4% in China [38,44,45,48], and 44.0% in Malaysia [31]. In the Peruvian study by Galea et al. [50], participants had little or no awareness of PrEP. In addition, the Indian study by Chakrapani et al. [52] reported that none of the participants in their study were aware of PrEP prior to the study. The proportions were highly heterogeneous (Q statistic = 2898, I^2^ = 99.5; (95% CI: 99.5–99.6), *p *< 0.001). Meta-analysis of the 13 studies that reported quantitative data of proportions of MSM who were aware of PrEP found that the pooled estimate of awareness among MSM was 29.7% (95% CI: 16.9–44.3) (Figure 3).

Besides overall awareness, few studies explored factors that were associated with awareness of PrEP. Older age in Brazil [39], more years of education in Brazil and Thailand [39,43], urban residence in China [45], frequent use of internet as a source of information in China [45], employment in Thailand [43], and non-local ethnicity in Thailand [43] were associated with higher levels of awareness. Two studies from Brazil [39] and China [45] reported conflicting results regarding the association between PrEP awareness and gender identity (gay versus TG or bisexual) or a recent STI diagnosis. Marital status was not associated with PrEP awareness in China [45].

However, the reported awareness did not necessarily reflect an accurate understanding of PrEP. Three studies checked whether the self-reported understanding of PrEP was accurate. In India, four participants who initially reported that they had heard of PrEP were later found to have mistaken PEP for PrEP [52]. In a Chinese study by Xia et al. [45], 19.1% of participants were aware of PrEP. However, when their self-reported understanding of PrEP was assessed, only around half of them (9.5%) had what could be considered an accurate understanding. Two studies [35,46] made a distinction between participants who were aware of the basics and those who fully understood PrEP and found that roughly half of participants who reported being aware of it had just a basic understanding.

In addition, some studies [33,34,50,52] provided information and definitions of PrEP *before* the assessment of awareness while others [32,49] defined it *after* assessment of awareness *but* before assessment of willingness to use it, which may have introduced varying potential for recall bias. This was compounded by the fact that participants had opportunities to ask clarification questions in studies that utilized face to face data collection methods [32,34,49,50,52], but did not have this opportunity in studies that used online methods exclusively [31,35,43]. Nevertheless, four studies provided useful information regarding sources of PrEP information, noting that participants may have heard of PrEP from the internet and print media in Malaysia and China [31,45], friends in Thailand, Malaysia, and China [31,45,47], healthcare providers in Thailand and Malaysia [31,47], or from previously publicized clinical trials in Peru [50].

## Willingness to use PrEP

**Figure 4.**
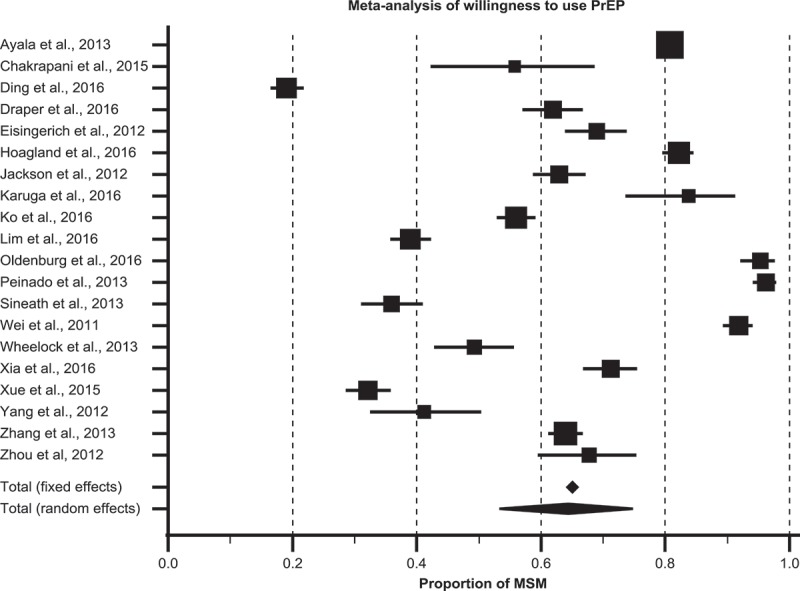
Pooled estimate of willingness to use PrEP among MSM in low- and middle-income countries.

Twenty studies assessed quantitative proportions of participants who were willing to use PrEP, while two studies reported qualitative assessment of willingness to use. Quantitative studies reporting willingness to use PrEP used a variety of scales and methods to derive the proportion of participants who were willing to use PrEP. Most studies [31,33–35,37,39–41,45,47,53], used different iterations of ratings on Likert-like scales, while a few [36,43,48,51] used simple binary measures. In general, proportions of participants willing to use PrEP varied significantly, ranging from a low proportion of 19.1% in China to a high proportion of 96.2% in Peru. Six studies reported low levels of willingness to use, in which less than half the participants were willing to use PrEP. These included 19.1% and 32.1% in two Chinese studies [36,46], 36%, 39.2% to 49.2% and 41% in three Thai studies [33,43,47], and 39.0% in Malaysia [31]. However, the majority of studies reported moderate-to-high levels of willingness to use PrEP. In nine studies, 50–70% of participants were willing to use PrEP, including 56.0% in Taiwan [40], 55.7% in India [52], 62% in Myanmar [37], 63.0–91.9% in China [44,48,49,53], and an average of 69.0% across India, Peru, and South Africa in a multi-country study [34]. In five studies, this proportion was >80%, and included 80.8% among LMIC participants in the multi-country study by Ayala et al. [35], 82.1% in Brazil [39], 83.3% in Kenya [51], 95.4% in Vietnam [41], and 96.2% in Peru [42]. As might be expected, these studies were highly heterogeneous (Q statistic = 3305, I^2^ = 99.4; (95% CI: 99.3–99.5, *p *< 0.001). Meta-analysis of these studies found that 64.4% (95% CI: 53.3–74.8) of MSM were willing to use PrEP (Figure 4).

## Factors associated with willingness to use PrEP

Table 5 illustrates the range of factors influencing MSM’s willingness to use PrEP documented in the included studies. These factors, which could potentially prevent or facilitate participants’ willingness to use of PrEP, conceptually fell into different categories within the individual, social (including partners, families, and communities) and structural domains (health systems and legal factors).

**Table 5.** Factors affecting willingness to use HIV pre-exposure prophylaxis (PrEP) among men who have sex with men in low- and middle-income countries

Domains	Barriers	Source study	Facilitating factors	Source study
Individual factors	Lack of PrEP information and awareness.	[37,39,43,45,48,50,52]	PrEP awareness and motivation to stay HIV negative.	[32,51,52]
	Concerns/doubts about PrEP effectiveness.	[31,32,43,44,49,52]	Perception that PrEP is 100% effective.	[50,51]
	Fear of side effects.	[31–33,44,49,51,52]	Need for intimacy and romance with a partner who is HIV positive.	[52]
	Low-risk perception among those at high risk	[32,46]	Multiple anal sex partners or history of STI or PEP.	[36,37,40,48]
	Need to take medicines frequently/daily.	[31,32,43,51]	Convenient dosing (injectable, monthly, or weekly).	[33,50]
	Competing preference for condoms (which can also be physically felt during sex).	[31,32,36]	Peace of mind if condom breaks or slips (PrEP as a second layer of protection).	[32,52]
Social factors	Fear of HIV stigma (since ARVs are used for treatment of HIV-positive people).	[49,51–53]	Ability to take PrEP pill discretely.	[52]
	Stigma towards homosexual orientation	[32,35,50,52]	Desire to protect sexual partner	[51]
	PrEP stigma or embarrassment using PrEP.	[31–33,35]	Peer and partner support.	[48–50,52]
Structural factors	Perceived attitudes of healthcare staff.	[50]	Wide availability of PrEP (clinics, community organizations, pharmacies, internet, etc.)	[31,32,50,52]
	Perceived lack of quality assurance.	[52]		
	Perceived lack of data confidentiality.	[32]	Discrete packaging.	[51]
	Cost.	[31,32,37,40,52]	Free or heavily subsidized PrEP.	[32,37,51]

## Individual factors

### Awareness, knowledge, and information about PrEP

Awareness is an important pre-requisite of utilization of health products, especially new interventions such as PrEP. In most studies, initial awareness of PrEP was low. However, once participants became aware of PrEP, most expressed interest in using it. For instance, although none of the participants were aware of PrEP in an Indian study by Chakrapani et al. [52], 55.7% of them reported willing to use it once they were informed of the concept of PrEP, its benefits for HIV prevention, and side effects. In Myanmar [37], 5.0% of the participants were initially aware, but 62.0% were willing to use it once they were informed about it. In China [48], awareness was 22.0% while willingness to use after introduction of the concept to the participants was 64.0%. In another Chinese study by Xia et al. [45], 19.1% were initially aware, but 71.3% were willing to use it once they became aware of it, and in yet another Chinese study [49], 11.2% were aware of PrEP, while 67.8% were willing to “definitely” or “probably” take PrEP if available. The same pattern was observed in Thailand [43], where 7.0% were aware and 36.0% were willing to use after PrEP was described, and in Brazil where 61.3% were aware and 82.1% were willing to use it [39]. In the multi-country study by Ayala et al. [35], awareness of PrEP was 69.8% while willingness to use it was 80.8% among participants from LMIC.

**Figure 5.**
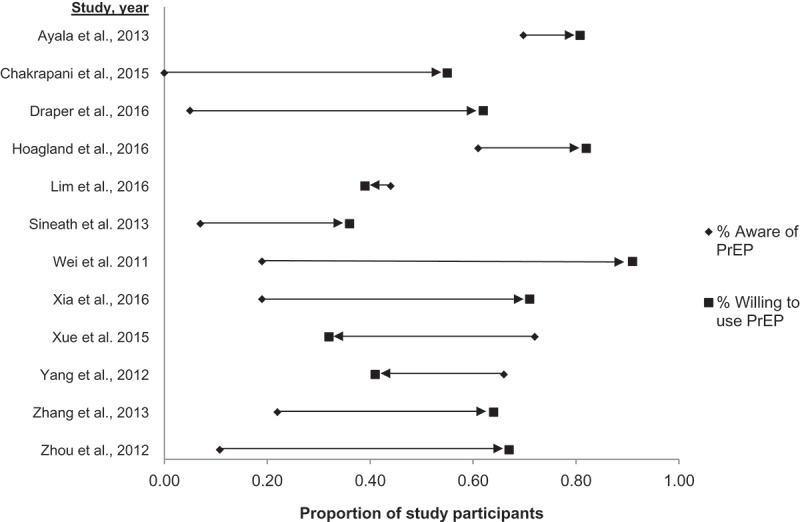
Relationship between awareness and willingness to use PrEP in studies reporting both outcomes.

However, three studies were exceptions to this general trend. In a Chinese study by Xue et al. [46], awareness was 72.8%, while willingness to use was much lower at 32.1%. In another Chinese study by Yang et al. [47], 66.0% were aware of PrEP, but 41.0% were willing to use it, while in the Malaysian study by Lim et al. [31], 44.0% were aware, but 39.0% were willing to use it (Figure 5).

### Motivation to stay healthy and HIV negative

Participants from Kenya identified provision of more information on PrEP as a factor that would facilitate their uptake and utilization of PrEP as they would have known of its preventive benefits [51]. In this study, the need to stay HIV negative and to protect their partners were common motivators for taking PrEP. In a typical response, one participant in a Peruvian study [50] elaborated his understanding and the reason he would take PrEP as about caring for his own health:

‘I [would] take PrEP, I would say that it is my own caring about myself, something which is only mine.’

This motivation regarding the protection of one’s own health was still sufficient even if participants had to pay for it, as explained by another MSM participant in the same study:

‘Yes, of course [I would pay for PrEP] … something that says that at least I am paying some of my own money for my health.’

### Fear of side effects

The most frequently explored determinant of potential PrEP use was perceived side effects. In most studies, such as in India [52], Thailand [33], Myanmar [37], Malaysia [31,32], Kenya [51], and China [44,49], participants had concerns about side effects, which were generally non-specific. In a study in China [49], 44.7% of MSM participants expressed specific worries regarding the impact of PrEP on their diet and sleep, while in Myanmar [37], participants were more concerned about long-term use. In the Kenyan study [51], and in response to whether he would use PrEP, one focus group discussion participant said that: “*I will not use it, because I don**’**t know if it will cause some harm in my body*.**”**

Although concerns regarding ARV side effects reduced willingness to use PrEP in most studies, it did not eliminate motivations to take it entirely. In Thailand [33], concern for side effects reduced the proportion of those willing to use PrEP. Before participants were informed about potential side effects, 39.2% were definitely willing to use PrEP, but 24.6% were still willing to take PrEP once they learnt about its possible side effects. A similar pattern was observed in the multi-country study by Eisingerich et al. [34] as shown in the above Table 2. In Vietnam [41], the proportion willing to use PrEP daily reduced from 95.4% to 56.7% given side effects.

### Need to adhere to PrEP

Three studies reported the requirement to take PrEP frequently as a barrier to future use of PrEP. In Thailand [43], concerns were reported about the need for daily dose as a potential barrier. In Kenya, participants singled out a general dislike of medicine as a deterrent [51]. Participants in Malaysia were aware of the need to adhere, but most admitted that they may “*lack the discipline to take PrEP on daily basis*” [32]. In another Malaysian study [31], 8.3% of the 603 participants who were not willing to use PrEP were worried about forgetting to take medication. Participants in the Thai study by Wheelock et al. [33] suggested that monthly injection in the arm could facilitate PrEP use by reducing the need for swallowing daily pills. In response to the question about ideal characteristics of PrEP, one MSM participant in a study in Peru [50] responded by saying that:

‘If they ask me to choose, I’d rather have it weekly or twice a week, by tablet, capsule, shot or whatever, it is far more likely than doing it daily.’

However, the preference for injections was not universal in all studies. In the multi-country study by Eisingerich et al. [34], Indian and Peruvian MSM preferred bimonthly injection in the buttocks, while South African MSM preferred daily pills to injection in the arm. In Vietnam [41], 27.7% of those who were willing to use PrEP preferred a lubricant to a pill. In contrast to the above studies, however, the Vietnamese study did not explore preferences regarding injectable PrEP.

### Risk perception

Psychological factors such as risk perception emerged as an important determinant of willingness to use PrEP. On the one hand, studies by Ding et al. [36] and Zhang et al. [48] found that MSM who had more anal sexual partners and those who had STIs were more willing to use PrEP. In Myanmar [37], willingness to use PrEP was higher among participants who had more than one regular partner (adjusted odds ratio (AOR):2.94; 95% confidence interval (CI) = 1.41–6.14) or more than five casual partners (AOR:2.05; 95% CI = 1.06–3.99). In this study, MSM who never or only occasionally used condoms with casual partners were more likely to be willing to use PrEP (AOR: 2.02; 95% CI = 1.00–4.10) in Taiwan [40], willingness to use PrEP was significantly associated with the previous receipt of HIV PEP (AOR:3.02, 95% CI = 1.49–6.12, *p *= 0.002). On the other hand, participants who perceived their risk of HIV to be low were unlikely to use PrEP. In a Malaysian study by Bourne et al. [32], some participants saw no need for PrEP as they were in monogamous relationships in which they used condoms, as they believed their risk was low. Several of these MSM also expressed that they would consider PrEP in the future if they would have a higher number of concurrent sexual partners. In another Malaysian study [31], 11.4% of the participants who were not willing to use PrEP identified the fact that they always used a condom as a reason why they would not need PrEP, implying that they thought that their risk of HIV was low. In Taiwan, participants who had sought and used HIV non-occupational PEP were significantly more likely to be willing to use PrEP [40]. In a Chinese study by Xue et al. [46], 54.2% of the participants did not want to use PrEP due to low self-risk assessment. In that study, 59.0% of participants perceived that “*risk behaviors were not happening every day*” and therefore they would not use PrEP. This sentiment was also reported in a Peruvian study by Galea et al. [50], where a participant expressed the following:

‘Well, if I am a person who has continuous [sexual] relationships yes, I’d take it, but if I [didn’t], why would I take it?’

However, participants’ perception of risk may not have been accurate. This lack of accurate risk perception may contribute to the paradoxical observation that participants in a Chinese study [48] who did not or rarely found sexual partners on the internet were more likely to be willing to use PrEP compared with higher-risk participants, who often or sometimes found sexual partners on the internet.

### Demographic factors

Eleven studies examined the association between willingness to use PrEP and a range of demographic factors. The association between participants’ demographic characteristics and willingness to use PrEP was generally inconsistent. Older age was found to be associated with willingness to use PrEP in two studies from China [36,49] and one study from Thailand [47]. However, this association was not found in studies in Kenya [51], Malaysia [31], Brazil [39], Taiwan [40], or in another study from China [48]. While two Chinese studies [45,48] suggested that willingness to use was higher among married participants as compared to unmarried, divorced, or widowed participants, three other studies from China [36,49] and Kenya [51] did not find such association. Several studies found that income [31,49] or employment status [31,40] were not associated with willingness to use PrEP [31]. In contrast, two Chinese studies [48,53] found that participants with lower monthly incomes were more willing to use it compared to those with higher monthly incomes.

Although two studies from China and Kenya suggested that bisexual participants were more likely to use PrEP compared to participants who identified themselves as homosexual [45,51], three studies in China [36,48,49] and one study in Brazil [39] did not find such association. Participants who had immigrated to the cities where the studies were conducted were reported to be more willing to use PrEP in Shanghai, China [36], but this association was not found in another study in Beijing, China [49] or Kuala Lumpur, Malaysia [31]. In addition, two studies examined the impact of rural versus urban residency reported contrasting results [48,53]. In the multi-country study by Ayala et al. [35], participants in LMIC expressed higher willingness to use PrEP compared to those from high-income countries. Local ethnicity was reported to be associated with willingness to use PrEP in Malaysia [31], although this was not commonly explored across other studies. One study reported an association between willingness to use PrEP and depressive symptoms among Chinese participants [53], but no other study reported association with mental health status. Overall, the evidence was inconsistent in regards to demographic factors.

### Uncertainty regarding the benefit of PrEP

Doubts regarding the benefit of PrEP were reported in Thailand, Malaysia, and China [32,43,44,49,52]. In China, 44.1% of participants expressed worries that PrEP had no prevention efficacy [49]. In Malaysia [31], 9.8% feared that PrEP would not work. Participants in qualitative studies in Malaysia and India thought that although PrEP has been shown to be effective in other high-income countries, it may not work well among local Asian MSM [32,52]. These concerns were particularly brought to the fore given that condoms were considered an alternative to PrEP by participants in Malaysia. In Thailand, 35% of MSM believed condoms were more effective than PrEP and therefore would prefer to use the former [43].

In Peru and Kenya, participants emphasized that being confident that PrEP is effective would motivate them to use it. A participant in a Peruvian study said: “*It would have to be 100% effective”* for him to use it [50], while in Kenya, a focus group participant stressed that: “*If I am sure it is going to work, it will motivate me to take it*.” [51]. However, 44.4% of participants in Taiwan [40] were willing to take PrEP even if it was not 100% effective, which may be related to the high likelihood of this study’s participants to use condoms: the majority of men willing to use PrEP indicated that they would maintain their condom use if taking PrEP (73.6% vs. 23.6%; *p *< 0.001).

### Preference for condoms

Concerns of effectiveness aside, findings suggested that some participants regarded condoms as mutually exclusive, and in this context, some MSM preferred condoms instead of PrEP. In Malaysia [31], 11.4% of the participants who reported not willing to use PrEP, identified the fact that they always use a condom as a reason why they would not need PrEP. In another Malaysian study [32], the physical barrier of condom was preferred to use drugs which cannot be seen and felt. One participant in this study remarked that “*there is a physical barrier that we can see in the condom, rather than drugs.”* In the Chinese study by Ding et al. [36], participants reporting condom use during their last anal sex with a man were significantly less willing to use PrEP (AOR:0.68; 95% CI = 0.47–0.97, *p *= 0.034), which may be linked to preference or risk perception.

This finding is particularly relevant given that MSM participants in a Peruvian study [50], and one key informant in an Indian study [52] were concerned that availability of PrEP could reduce the use of condoms. One Peruvian MSM participant, in response to a question about the impact of PrEP, responded by saying that:

‘If you tell someone, “Look, take this pill and it will prevent you from getting HIV,” I can assure you that the next day, that person won’t use a condom anymore.’

In a typical response regarding how to deal with this situation, participants in this Peruvian study suggested the need for PrEP education to clarify if and how condoms should be used in combination with PrEP, while emphasizing that PrEP may not be 100% effective:

‘There should be a lot of information and say that it is something additional to condoms and which is going to give you some extra protection. If you tell them that [PrEP] is 100% protective, they won’t use [a condom] anymore.’

### PrEP as a back-up plan

In Malaysia and India, there were participants who considered PrEP a complementary strategy in the context of inconsistent condom use [52], or as a “second layer” of protection in case condoms fail [32], or on occasions where condom use was intended, but did not occur.

### Potential resistance

One study reported concerns regarding the potential emergence of ARV drug resistance. In a Chinese study [49], 21.7% of participants expressed worries about ARV drug resistance from PrEP, and saw resistance as an important factor to consider while making decision whether to use PrEP.

### Need for frequent monitoring and testing

Data from two studies reported the influence that the need for frequent clinical monitoring and HIV testing could have on individual willingness to use PrEP. In a multi-country study by Eisingerich et al. [34], 55.0–88.0% were willing to use PrEP with regular HIV testing. However, a study in Thailand [43] indicated that overall, 28.0% of participants “*didn’t want to see a doctor every three months*.” In Kenya [51], a minority of participants also reported they would not want to return for the required regular HIV testing, and one asserted that: “*I will visit the clinic to see if it will work, but after that, if it works, I will rarely visit clinic, will just continue to use the medicine*.”

## Social factors

A range of factors in family and community domains were reported to influence willingness to use PrEP.

### HIV stigma

Because similar ARV drugs are used for PrEP as for treatment of those infected with HIV, HIV-related stigma, specifically fear of being mistakenly identified as a person with HIV [49,51], or being identified as a person at risk of HIV [52,53] were identified as potential barriers of the future use of PrEP. Fear of being identified as a person at risk of HIV was particularly prevalent in India and China [52,53]. In one Chinese study [49], 20.1% of MSM participants expressed worries about using PrEP for fear of being “*treated as an AIDS patient by people.”* Participants from Kenya suggested that using different packaging from that used for ARV drugs for treatment of HIV could reduce potential perceived HIV-related stigma [51]. Participants stressed that unless that was done, peers would begin “*classifying you as HIV positive*.”

### Stigmatization of PrEP and homosexual orientation

Apart from HIV-related stigma, three studies documented potential stigma that can be associated with users of PrEP, based on their assumed sexual orientation and behaviours. In some studies, this phenomenon was referred as “PrEP stigma” [35]. In a Malaysian study [32], some participants felt that being on PrEP would be perceived by peers as having a direct association with riskier behaviours such as *barebacking* or using drugs during sex. In this study [32], PrEP was linked to sex work by a participant who suggested that PrEP use is “*a money boy or go-go boy who is not in a position to negotiate safe sex*.” In a Peruvian study [50], MSM were wary of accessing PrEP in neighbourhood drugstores because they were “*afraid of being identified as a person who has* [homo] *sexual relationships.”* In an Indian qualitative study [52], beliefs that potential PrEP users were high-risk individuals – promiscuous, sex workers, or have multiple sex partners – was also reported as a potential barrier to the uptake, due to anticipated stigma. In the multi-country study by Ayala et al. [35], PrEP stigma was negatively correlated with willingness to use it (β: −0.51; 95% CI = − 0.55 to −0.48, *p *< 0.001). These themes were further advanced by a study in Thailand [33] that explored the extent to which participants may feel embarrassed to use PrEP, and found that 2.7% thought that taking PrEP would potentially be “very embarrassing” and 5.8% “fairly embarrassing.” In another Malaysian study [31], of the participants who reported not willing to use PrEP, 5.8% identified concerns about *“what other people might think of me”* as a reason they would not use it.

### Importance of partner, peer, and family support

Data from three studies suggested that influence, reaction, or support from partners, peers and family could either be a barrier or facilitator of PrEP use. In an Indian qualitative study, the possibility of covert use of PrEP not requiring partner approval was seen as a facilitating factor [52], suggesting that some participants fear that their partners may not be supportive. In a Chinese study [49], 14.5% of participants were worried about being refused sex by male partners after using PrEP. In a multi-country study by Eisingerich et al. [34], proportions of participants who would definitely want their partner or partners to know if they were taking PrEP were 70.0% among Indian, 52.0% among Peruvian, and 68.0% among South African participants. The importance of peer influence was reported in China [48], where the proportion willing to use PrEP increased from 64.0% to 77.0% if it were free and also used by people known to the participants. In Peru, the influence of peers was also documented [50]. In a typical response characterizing other participants in the study, one MSM reported that he would consider using PrEP because “*most of my friends were going to use it*.” In this Peruvian study [50], participants also expressed concern regarding potential judgment from family if he was discovered to be using PrEP:

‘I think that there would be some kind of rejection from my family…they would think I am a promiscuous person.’

## Structural factors

### Cost of PrEP

Cost emerged as an important barrier to use of PrEP in India, China, Kenya, Malaysia, Peru, Myanmar and Taiwan. In India, participants anticipated drugs for PrEP to be highly priced [52]. In China, 26.3% of participants expressed worry about not being able to afford PrEP [49]. In Malaysia, participants suggested that PrEP should be free of charge at the point of access, or at a reduced cost, with the government covering the cost [31,32]. In this context [31], 8.8% of the participants who were not willing to use PrEP identified failure to afford it as the reason they would not use it. In the Chinese study by Zhang et al. [48], the proportion of MSM willing to use PrEP increased from 64.0% to 71.0% if it were completely free. In Myanmar, willingness to use PrEP was 62% as long as participants were not required to pay for it [37]. In Peru [50], however, participants noted that if initially provided free of charge, users should not be required to pay for it later as that approach may reduce its utilization:

‘If free…they would get used to have it for free all the time, and when it is unavailable, they just won’t buy it.’

In Kenya, participants in focus group discussions suggested that they would be motivated to use PrEP if it would be available at a subsidized cost to a price comparable to that of condoms [51]. Subsidized or free distribution of PrEP through community-based organizations was also identified as a potential facilitator of PrEP uptake among lower socioeconomic status MSM in India [52].

In most studies, having to pay for PrEP reduced but did not eliminate willingness to use it. In Malaysia, about one-third (35.6%) of participants were willing to pay out-of-pocket for PrEP, and of these, 88% were willing to pay for it if it cost less than RM200 (USD50) per month [31]. In Taiwan, 56.0% participants initially expressed willingness to use PrEP, but the percentage fell to 23.0% when participants were asked if they were willing to pay an estimated USD340 monthly for it [40]. Similarly, in Brazil [39], 75.8% of all participants reported that they would use PrEP even if they had to pay for it, a reduction from an overall rate of 82.1%. In a multi-country study by Eisingerich et al. [34], 39.0–88.0% of participants were willing to use PrEP despite having to pay for it. In a Thai study [43], 65.0% of those willing to use PrEP (36.0%) indicated they would be willing to pay for it. In another Thai study [33], 58.8% were “definitely” while 35.0% were “probably” willing to use PrEP despite having to pay 500 Baht (USD15) a month for it.

### Access to and attitudes of health professionals

Data from one study in Vietnam [41] indicated that previous contact with peer health educators doubled the odds of willingness to use PrEP (AOR: 2.28; 95% CI = 1.25–4.14, *p *< 0.05). Findings from Peru emphasized the importance of health-care professionals, especially in relation to stigmatizing attitudes [50]. In the multi-country study by Ayala et al. [35], participants were concerned about potential stigmatizing attitudes from health providers. However, findings from this study suggested that having experienced stigma was positively correlated with higher willingness to use of PrEP (β: 0.12; 95% CI = 0.02–0.23, *p *= 0.021), probably because PrEP could be used without having to access healthcare facilities for other HIV prevention services.

### Confidentiality and data protection

The fear of poor confidentiality and lack of data protection of MSM’s identity, especially in public health facilities, was identified as a barrier in a study in Malaysia [32], where a participant remarked that “*in the government clinic you have to register, it has to be on record, so that’s not discreet.”* This was particularly important for participants given stigma and cultural and religious sentiments towards MSM in the Malaysian context.

### Quality assurance of PrEP

A belief that pharmacies may distribute fake PrEP undermined trust in the quality and potency of PrEP in India [52]. Although quality can affect the effectiveness of PrEP in any context, these concerns seemed unique to pharmaceutical quality control systems in India, as they were absent in other studies.

### Ways to access PrEP

Several studies explored ways in which MSM wanted to access PrEP. Venues identified for potential PrEP access included public health facilities [31,50,52], community-based organizations (CBOs) [31,32,52], pharmacies [31,50], and online websites [31]. Participants’ preferences were not consistent, but depended on their perceptions related to stigma, data protection, and costs at each of these venues. In India [52], some participants preferred PrEP to be provided through government facilities as they were concerned that if dispensed through CBOs, other MSM might find out and label them as promiscuous. In contrast, government facilities were viewed as likely to compromise data confidentiality in Malaysia, and CBOs were preferred instead [32]. MSM in Peru [50] preferred PrEP being available in healthcare centres as opposed to pharmacies, citing higher costs. Overall, these findings suggest that increasing ways through which PrEP can be provided could increase its uptake, as it would cater for the needs and preferences of different MSM.

## Discussion

This review set out to determine the awareness of and willingness to use PrEP among MSM in low-and middle-income countries. The review found that although it varies, awareness of PrEP among MSM in low-and middle-income countries is generally low, ranging from 0% to 72%, with a pooled awareness of 29.7% (95% CI: 16.9–44.3) across all studies. In contrast, willingness to use PrEP is relatively high, ranging from 19% to 96%, with a pooled estimate of 64.4% (95% CI: 53.3–74.8). These results suggest that once MSM become aware of PrEP, the majority are willing to use it.

The finding of low PrEP awareness echoes that from a previous review by Young and McDaid which showed that MSM, sex workers, injecting drug users, and serodiscordant participants in PrEP studies that were not part of larger PrEP clinical research trials had limited knowledge of PrEP, ranging from 11.0 % to 23.0 % [15]. Our review suggests that actual awareness of PrEP may be lower than reported in previous studies, given that participants’ understanding of PrEP was not accurate in two studies [45,52]. This has also been observed in studies from high-income countries. In a US study [56] in which 62.0 % of participants had claimed to have heard of PrEP, one-quarter were found to have mistook PEP for PrEP.

Compared to studies in high-income settings, this review suggests that willingness to use PrEP is relatively higher among MSM in low-and middle-income countries. A 2012 study reported that only 28.2% of Australian MSM were willing to use PrEP [57]. More recently, in Europe, proportions of MSM willing to use PrEP have ranged from 47.8% to 54.3% in Scotland [58,59], 57.0% in Portugal [60] and 57.6 % in Spain [61]. Recent Canadian studies reported willingness to use PrEP of 55.0% [62,63]. In the US where the majority of studies on PrEP have been conducted, willingness to use PrEP has ranged from 46.1% to 71.0% [64–69].

Nevertheless, the reported willingness to use could eventually change when PrEP is actually offered as was the case in one Chinese study [36] included in this review, whereby 20.5% changed their minds when PrEP was subsequently offered. The accuracy with which willingness to use predicts actual acceptance is difficult to determine as it could change based on individual circumstances as well as the setting within which PrEP is provided. For instance, the majority of MSM in open-label extensions of RCTs show willingness to enrol, and most go on to use it [6,70–72], although with sub-optimal adherence [71,72]. However, contexts of open-label extensions and preceding trials differ from regular programmes in terms of patient preparedness, education and support, and may therefore positively bias its use [15]. More realistic observations from 20 US cities suggested that although over half of MSM reported willing to take PrEP, only 4% actually used it [68]. Therefore, understanding the relationship between willingness to use and actual uptake of PrEP in “real-world” HIV prevention programmes in LMIC should be prioritized in future PrEP implementation research.

Besides the overall willingness to use PrEP, our review provides important information regarding barriers and motivations of its use, in response to earlier calls to increase understanding of the context within which PrEP might be accessed and utilized [15]. In particular, this review identified a range of individual, social and structural factors that may influence the willingness to use PrEP. In the individual domain, poor knowledge about PrEP, and doubts about its effectiveness, were common potential barriers, alongside fear of side effects, low-risk perception among those at high risk, and inconvenience of having to ingest medicines daily. In addition, participants tended to view PrEP as a competing intervention against condoms.

These findings suggest that PrEP education and information should be prioritized, including provision of accurate information about the role of PrEP within combination HIV prevention as recommended by WHO [4,5]. Differentiating PrEP from PEP to potential adopters in LMIC would be important, at least initially. Studies from high-income settings such as Spain [61] have demonstrated the importance of awareness in determining willingness to use PrEP. Beyond just hearing about PrEP, the way in which PrEP information is provided to MSM, and how well they were able to understand it, could influence their levels of willingness to use it. In this review, some studies provided opportunities for one-to-one discussions on PrEP, others provided this information through anonymous survey, and while each has its merits, they could influence willingness differently. Our review also found that peers, healthcare providers, print media, and internet websites are all useful sources of information about PrEP for MSM. Provision of PrEP and HIV prevention information through online and mobile dating applications could be particularly effective in reaching MSM at higher risk of HIV infection. This is particularly relevant given that high-risk MSM in at least two studies did not always view themselves to be at risk of HIV [32,48].

HIV risk perception and behaviours have been found to be important determining PrEP utilization in high-income settings [12,62,73]. Four studies in this review [36,37,40,48] found that MSM who had more anal or casual sexual partners, those who had received PEP, and those with a history of STIs were more willing to use PrEP, possibly because they had stronger perceptions of risk. A recent review found that PrEP use was associated with STIs among MSM [74], which underscores the need for comprehensive prevention package [75]. However in our review, it was notable that willingness to use PrEP was high among some participants who were at low risk e.g. those who had one regular partner or no casual partners in Myanmar [37], which could be due to incorrect risk perception, or bias to use HIV prevention among MSM who were already practising safe sex behaviours. Disconnect between objective and subjective HIV risk has also been identified in MSM from high-income settings [76]. To address these uncertainties of risk and optimize PrEP utilization among those at substantial risk of HIV as recommended by WHO [4,5], additional research and programmatic tools should be devised to assist individuals have a better self-awareness of their HIV risk.

In addition, it is critical to continue exploring ways in which convenient and safe PrEP can be delivered to sidestep concerns regarding the need for frequent ingestion of drugs. Concerns regarding long-term adherence have also been reported in the UK as a deterrent of potential PrEP use [12]. In our review, injectable or less frequent dosage schedules were more preferable to oral pills [33,34,43,50]. However, user preferences are highly contextual. In the US, a study reported that MSM preferred daily oral pills and non-visible implants over injections or visible implants, citing convenience, duration of protection, and privacy [77]. A recent trial found that PrEP adherence was higher in daily, compared to less frequent time- or event-driven dosing regimens [78], although participants in this trial were simply randomized to these arms and didn’t have a choice. While conforming to user preferences is essential for a tailored PrEP programme, method of delivery may have an effect on cost, adherence, PrEP coverage per sex-act, and ultimately, “real-world” effectiveness.

A range of other individual demographic factors including educational level, age, and residency were noted to influence willingness to use PrEP. However, these were not universal nor were their impacts consistent across all studies. However, there is a need to tailor provision of PrEP to specific MSM who are particularly at risk of HIV based on their age groups, migration and socio-economic status and other characteristics, as part of effectively tailored combination of HIV prevention  interventions. For instance, studies from the USA suggest that PrEP can be feasibly provided to young MSM [79].

Within social and interpersonal domains, stigma was the single most commonly encountered factor. Our review found that MSM anticipate stigma from peers, partners, family as well as healthcare providers and that this stigma may be either related to HIV [49,51–53], or behaviours that may warrant the use of PrEP [80,81], such as sex work [32] and homosexual sex [31–33,35,50,52]. These findings suggest that it is essential to integrate strategies to mitigate stigma related to sexual orientation as well as HIV within PrEP programming. Chakrapani et al. suggest that community engagement may facilitate broad acceptability and challenge stigma around PrEP [52]. Unfortunately, existing evidence suggests that although community mobilization and collectivization interventions can indeed mitigate stigma [82], it is difficult to eliminate it unless these social interventions are combined with structural interventions and this may include decriminalization of same-sex relations in some settings.

Stigmatization in health facilities is particularly detrimental as it can prevent health seeking for other services, suggesting that competency and skill-based training, sensitization, and performance improvement to enable provision of friendly HIV prevention services will be required, as argued by others [81]. The extent of partner, peer, and family support was a significant factor affecting willingness to use PrEP, especially in the context of stigma. Therefore, PrEP programmes will need to ensure that consideration is given about how partners and peers of MSM can be leveraged on to facilitate, rather than hinder, access and utilization of PrEP.

Key structural factors included perceived staff attitudes, a lack of quality assurance, a lack of data protection and confidentiality, and cost [31,32,37,40,50,52]. Apart from addressing stigma and general competency to provide MSM with PrEP among healthcare providers in LMIC – which have been noted by others [83,84] – our results suggest that strengthening health systems so as to assure universal provision of high-quality PrEP, while protecting the identities of MSM, will be critical. Strengthening drug regulation in middle-income countries has also been highlighted as essential by other authors [85]. In this review, cost was a common determinant of willingness to use PrEP. Although several studies suggested thresholds at which MSM would be willing to pay for PrEP, the studies were conducted in diverse LMICs and socio-economic backgrounds, making it difficult to generalize thresholds. Furthermore, most studies were conducted in cities, and mostly in middle-, rather than low-income countries. Nevertheless several studies [31,33,34,39,40,43,48,51] suggested that there were MSM in Kenya, Malaysia, Brazil, Taiwan, Thailand, Peru, India, and South Africa who might be motivated to use PrEP, despite having to pay full or subsidized price for it.

## Strengths and limitations of this review

Our findings build on those of previous reviews conducted by Holt, Young, and McDaid [14,15] which focused on general acceptability of ARV-based prevention. A unique strength of our review is the focus on, and inclusion of, a substantially greater number of studies from low- and middle-income countries. Most studies in this review were Asian with relatively limited data from low- and middle-income countries in Eastern Europe, West and North Africa, or the Caribbean counties. However, inclusion of multi-country studies [34,35] strengthened the generalizability of this review to LMIC settings. In addition, the focus of the review on awareness and willingness to use realistically reflects the current early stages of PrEP introduction in LMIC, as it excludes acceptability which is more suited for high-income settings where PrEP for MSM is more widely available.

The range of individual, social, and structural factors identified in this study are likely to remain relevant even after PrEP among MSM becomes available. Sub-optimal uptake and retention in PrEP has been reported in the US outside of open-label extensions [86–88] often due to similar issues identified in this review such as poor awareness levels, cost, low risk perception, need for daily intake, and fear of potential side effects [87–89]. Addressing these barriers in low- and middle-income countries is particularly relevant given that implementation of PrEP in LMIC has lagged behind compared to high-income countries [15,16]. Rapidly scaling up access and utilization of PrEP will require mitigating barriers while accentuating facilitators of its potential uptake identified in this review.

We did not conduct sub-group analyses. Geographic and socio-demographic comparisons could provide insights into contextual determinants of awareness or willingness to use PrEP. As noted previously, reported proportions were highly heterogeneous. The diversity of scales and measures used to estimate willingness to use PrEP in the included studies may have affected the precision of our estimates, since the observed proportions might not be entirely attributed to sampling error, and other factors such as differences in MSM participants and their settings could also contribute. For this reason, we used random-effects model rather than fixed effects model, which provides more conservative estimates e.g. wider 95% CIs, while assuming that the measurement of the parameter of interest may not be entirely identical across studies [29]. Nevertheless, our overall findings are consistent with previous reviews which observed high rates of willingness to use PrEP, regardless of the type of scales used in individual studies [14,15].

Although all the included studies provided sufficient information to enable assessment of risk of bias, this was found to be relatively high, mainly due to study designs and necessary reliance on self-reported sexual behaviour data. Survey and interview questions related to sensitive, criminalized or taboo sexual activities often generate inaccurate estimates due to social desirability bias [90]. The perception of PrEP varied both between and within studies, with its use being seen as responsible by some participants [50] and as indicative of risky sexual behaviour by others [32,33,50,52]. Thus, it is not possible to state with certainty the net effect of social desirability bias on our overall findings.

Findings of this review are not generalizable beyond oral PrEP which was the focus herein; awareness and willingness to use other forms of PrEP might be different. Because PrEP is frequently explored in the context of expanding combination prevention options for populations at highest risk [4,85,91], awareness and willingness to use it could vary based on contact with PrEP-related research and demonstration projects [81,92]. Although several databases were searched, some studies may have been missed. Relevant information reported in conference abstracts and non-peer reviewed literature may have been missed as the review only considered peer-reviewed publications to minimize bias. While language limitations were not applied, no studies published in languages other than English and Chinese were found. Publishing bias may still exist. Nevertheless, this review utilized standard approaches of conducting [30] and reporting systematic reviews [17] to minimize bias.

## Conclusions

Over the last few years, RCTs have demonstrated the effectiveness of oral PrEP in reducing the risk of HIV acquisition among MSM [9,10], demonstration projects are increasingly being implemented [71,72,81] and the WHO has endorsed the use of PrEP by MSM and other populations at substantial risk of HIV [4,5]. Although PrEP policies are starting to be put into place in counties such as Malaysia, Kenya, and South Africa, actual implementation of PrEP in low- and middle-income countries has been relatively limited [15,16]. In addition, criminalization of same sex relations may limit uptake of prevention services among MSM [93]. Programmes intended to introduce or scale-up usage of PrEP need to be based on context-specific evidence, such as potential demand and user preferences, supported by enabling legal and policy framework environments. This review contributes to this evidence base by demonstrating that despite currently low awareness of PrEP, MSM in low- and middle-income countries are willing to use it once they become aware of it and they should be appropriately supported to deal with a range of individual, social, and structural barriers.

## Notes

To access the supplementary material to this article please see Supplementary Files under Article Tools online.

## References

[1] Murray CJ, Barber RM, Foreman KJ, Abbasoglu Ozgoren A, Abd-Allah F, Abera SF, et al Global, regional, and national disability-adjusted life years (DALYs) for 306 diseases and injuries and healthy life expectancy (HALE) for 188 countries, 1990-2013: quantifying the epidemiological transition. Lancet. 2015;386(10009):2145–27. doi:10.1016/s0140-6736(15)61340-x 26321261 PMC4673910

[2] Wang H, Wolock TM, Carter A, Nguyen G, Kyu HH, Gakidou E, et al Estimates of global, regional, and national incidence, prevalence, and mortality of HIV, 1980-2015: the Global Burden of Disease Study 2015. Lancet HIV. 2016;3(8):e361–87. doi:10.1016/s2352-3018(16)30087-x 27470028 PMC5056319

[3] UNAIDS Global AIDS update, 2016. Geneva: UNAIDS; 2016.

[4] WHO Guidance on Pre-Exposure Oral Prophylaxis (PrEP) for serodiscordant couples, men and transgender women who have sex with men at high risk of HIV. Recommendations for use in the context of demonstration projects. Geneva: World Health Organization; 2012. 23586123

[5] WHO Guideline on when to start antiretroviral therapy and on pre-exposure prophylaxis for HIV. Guideline on when to start antiretroviral therapy and on pre-exposure prophylaxis for HIV. Geneva: World Health Organization; 2015. 26598776

[6] Grant R, Lama J, Anderson P, McMahan V, Liu A, Vargas L, et al Preexposure chemoprophylaxis for HIV prevention in men who have sex with men. N Engl J Med. 2010;363(27):2587–99. doi:10.1056/NEJMoa1011205 21091279 PMC3079639

[7] Choopanya K, Martin M, Suntharasamai P, Sangkum U, Mock PA, Leethochawalit M, et al Antiretroviral prophylaxis for HIV infection in injecting drug users in Bangkok, Thailand (the Bangkok Tenofovir Study): a randomised, double-blind, placebo-controlled phase 3 trial. Lancet. 2013;381(9883):2083–90. doi:10.1016/s0140-6736(13)61127-7 23769234

[8] Baeten JM, Donnell D, Ndase P, Mugo NR, Campbell JD, Wangisi J, et al Antiretroviral prophylaxis for HIV prevention in heterosexual men and women. N Engl J Med. 2012;367(5):399–410. doi:10.1056/NEJMoa1108524 22784037 PMC3770474

[9] Molina J-M, Capitant C, Spire B, Pialoux G, Cotte L, Charreau I, et al On-demand preexposure prophylaxis in men at high risk for HIV-1 infection. N Engl J Med. 2015;373(23):2237–46. 26624850 10.1056/NEJMoa1506273

[10] McCormack S, Dt D, Desai M, Di D, Gafos M, Gilson R, et al Pre-exposure prophylaxis to prevent the acquisition of HIV-1 infection (PROUD): effectiveness results from the pilot phase of a pragmatic open-label randomised trial. Lancet. 2016;387(10013):53–60. doi:10.1016/S0140-6736(15)00056-2 26364263 PMC4700047

[11] Spinner CD, Boesecke C, Zink A, Jessen H, Stellbrink H-J, Rockstroh JK, et al HIV pre-exposure prophylaxis (PrEP): a review of current knowledge of oral systemic HIV PrEP in humans. Infection. 2016;44(2):151–58. 26471511 10.1007/s15010-015-0850-2

[12] Young I, Flowers P, McDaid LM. Barriers to uptake and use of pre-exposure prophylaxis (PrEP) among communities most affected by HIV in the UK: findings from a qualitative study in Scotland. BMJ Open. 2014;4(11):e005717. doi:10.1136/bmjopen-2014-005717 PMC4244494 25412863

[13] Rocha LM, Campos MJ, Brito J, Fuertes R, Rojas J, Pinto N, et al Acceptability of PrEP among HIV negative Portuguese men who have sex with men that attended 2014 Lisbon pride fair. J Int AIDS Soc. 2014;17(4 Suppl 3):19734. doi:10.7448/IAS.17.4.19734 25397480 PMC4225295

[14] Holt M HIV pre-exposure prophylaxis and treatment as prevention: a review of awareness and acceptability among men who have sex with men in the Asia-Pacific region and the Americas. Sex Health. 2014;11(2):166–70. doi:10.1071/SH13060 23866853

[15] Young I, McDaid L How acceptable are antiretrovirals for the prevention of sexually transmitted HIV? A review of research on the acceptability of oral pre-exposure prophylaxis and treatment as prevention. AIDS Behav. 2014;18(2):195–216. doi:10.1007/s10461-013-0560-7 23897125 PMC3905168

[16] Caceres CF, Bekker LG, Godfrey-Faussett P No one left behind: how are we doing in the roll-out of PrEP as part of combination HIV prevention? J Int AIDS Soc. 2016;19(7(Suppl6)):21364. doi:10.7448/ias.19.7.21364 27760690 PMC5071753

[17] Moher D, Liberati A, Tetzlaff J, Altman DG Preferred reporting items for systematic reviews and meta-analyses: the PRISMA statement. BMJ. 2009;339:b2535. doi:10.1136/bmj.b2535 19622551 PMC2714657

[18] Moher D, Shamseer L, Clarke M, Ghersi D, Liberati A, Petticrew M, et al Preferred reporting items for systematic review and meta-analysis protocols (PRISMA-P) 2015 statement. Syst Rev. 2015;4:1. doi:10.1186/2046-4053-4-1 25554246 PMC4320440

[19] Mays N, Pope C, Popay J Systematically reviewing qualitative and quantitative evidence to inform management and policy-making in the health field. J Health Serv Res Policy. 2005 7;10(Suppl 1):6–20. doi:10.1258/1355819054308576 16053580

[20] Stokols D Translating social ecological theory into guidelines for community health promotion. A J Health Promotion. 1996;10(4):282–98. 10.4278/0890-1171-10.4.282 10159709

[21] Mburu G, Ram M, Oxenham D, Haamujompa C, Iorpenda K, Ferguson L Responding to adolescents living with HIV in Zambia: a social–ecological approach. Child Youth Serv Rev. 2014;45:9–17.

[22] Busza J, Walker D, Hairston A, Gable A, Pitter C, Lee S, et al Community-based approaches for prevention of mother to child transmission in resource-poor settings: a social ecological review. J Int AIDS Soc. 2012;15(Suppl 2):17373. doi:10.7448/IAS.15.4.17373. PMC3499910 22789640

[23] Pope C, Mays N, Popay J How can we synthesize qualitative and quantitative evidence for healthcare policy-makers and managers? Healthc Manage Forum. 2006;19(1):27–31. 17330642 10.1016/S0840-4704(10)60079-8

[24] Howe K Mixed methods, triangulation, and causal explanation. J Mix Methods Res. 2012;6(2):89–96.

[25] Bryman A Social research methods. Oxford: Oxford University Press; 2012.

[26] Silverman D Interpreting qualitative data. Methods for analyzing talk text and inter- action. 2nd ed. London: Sage Publication; 2001.

[27] Charmaz K Grounded theory: objectivist and constructivist methods In: Denzin NK, Lincoln YS, editors. Handbook of qualitative research. Thousand Oaks, CA: Sage, 2000 p. 509–35.

[28] Strauss A, Corbin J Basics of qualitative research: techniques and procedures for developing grounded theory. 2nd ed. Thousand Oaks, CA: Sage; 1998.

[29] Nyaga VN, Arbyn M, Aerts M Metaprop: a Stata command to perform meta-analysis of binomial data. Arch Public Health. 2014;72(1):39. doi:10.1186/2049-3258-72-39 25810908 PMC4373114

[30] Green S, Higgins J, Alderson P, Clarke M, Mulrow C, Oxman A Cochrane handbook for systematic reviews of interventions, Vol. 5 Chichester, UK: Wiley-Blackwell; 2008.

[31] Lim SH, Mburu G, Bourne A, Pang J, Wei CKT, Wickersham, JA, et al. Willingness to use pre-exposure prophylaxis for HIV prevention among men who have sex with men in Malaysia: Findings from an online survey. Submitted 2016. 10.1371/journal.pone.0182838 PMC5597127 28902857

[32] Bourne A, Cassolato M, Wei CKT, Wang B, Pang J, Lim SH, et al. Willingness to use pre-exposure prophylaxis (PrEP) for HIV prevention among men who have sex with men (MSM) in Malaysia: findings from a qualitative study. Submitted 2016. 10.7448/IAS.20.1.21899 PMC5577697 28782336

[33] Wheelock A, Eisingerich AB, Ananworanich J, Gomez GB, Hallett TB, Dybul MR, et al Are Thai MSM willing to take PrEP for HIV prevention? An analysis of attitudes, preferences and acceptance. PLoS One. 2013;8(1):e54288. doi:10.1371/journal.pone.0054288 23342121 PMC3544831

[34] Eisingerich AB, Wheelock A, Gomez GB, Garnett GP, Dybul MR, Piot PK Attitudes and acceptance of oral and parenteral HIV preexposure prophylaxis among potential user groups: a multinational study. PLoS One. 2012;7(1):e28238. doi:10.1371/journal.pone.0028238 22247757 PMC3256136

[35] Ayala G, Makofane K, Santos GM, Beck J, Do TD, Hebert P, et al Access to basic HIV-related services and PrEP acceptability among men who have sex with men worldwide: barriers, facilitators, and implications for combination prevention. J Sex Transm Dis. 2013;2013:953123. doi:10.1155/2013/953123 26316968 PMC4437423

[36] Ding Y, Yan H, Ning Z, Cai X, Yang Y, Pan R, et al Low willingness and actual uptake of pre-exposure prophylaxis for HIV-1 prevention among men who have sex with men in Shanghai, China. Bioscience Trends. 2016;10(2):113–19. doi:10.5582/bst.2016.01035 27052151

[37] Draper B, Oo ZM, Thein ZW, Aung PP, Veronese V, Ryan C et al. Acceptability of HIV pre-exposure prophylaxis among gay, men other men who have sex with men, and transgender women in Mynamar. Submitted 2016. 10.7448/IAS.20.1.21885 PMC5577714 28741332

[38] He JT, Zhong XN, Lu J, Peng B, Zhang Y, Liang H, et al Propaganda methods of pre-exposure prophylactic medication in men who have sex with men: a multiple correspondence analysis. [Chinese] Acad J Sec Milit Med Uni. 2014;35(2):122–28. doi:10.3724/SP.J.1008.2014.00122

[39] Hoagland B, De Boni R, Moreira R, Madruga J, Kallas E, Goulart S, et al Awareness and Willingness to Use Pre-exposure Prophylaxis (PrEP) among men who have sex with men and transgender women in Brazil. AIDS Behav. 2016;21:1278. doi:10.1007/s10461-016-1516-5 [Epub ahead of print]. 27531461

[40] Ko NY, Chen BJ, Li CW, Ku WW, Hsu ST Willingness to self-pay for pre-exposure prophylaxis in men who have sex with men: a national online survey in Taiwan. AIDS Educ Prev. 2016;28(2):128–37. doi:10.1521/aeap.2016.28.2.128 27459164

[41] Oldenburg C, Le B, Huyen H, Thien D, Quan N, Biello K, et al Antiretroviral pre-exposure prophylaxis preferences among men who have sex with men in Vietnam: results from a nationwide cross-sectional survey. Sex Health. 2016 [Epub ahead of print]. doi:10.1071/SH15144 PMC5253341 27444753

[42] Peinado J, Lama JR, Galea JT, Segura P, Casapia M, Ortiz A, et al Acceptability of oral versus rectal HIV preexposure prophylaxis among men who have sex with men and transgender women in Peru. J Int Assoc Provid AIDS Care. 2013;12(4):278–83. doi:10.1177/1545109712473650 23422742

[43] Sineath RC, Finneran C, Sullivan P, Sanchez T, Smith DK, Griensven F, et al Knowledge of and interest in using preexposure prophylaxis for HIV prevention among men who have sex with men in Thailand. J Int Assoc Provid AIDS Care. 2013;12(4):227–31. doi:10.1177/2325957413488184 23708677

[44] Wei SS, Zou YF, Xu YF, Liu JJ, Nong QX, Bai Y, et al Acceptability and influencing factors of pre-exposure prophylaxis among men who have sex with men in Guangxi. [Chinese]. Zhonghua Liu Xing Bing Xue Za Zhi = Zhonghua Liuxingbingxue Zazhi. 2011;32(8):786–88. 22093468

[45] Xia W, Bourne A, Pulin L, Jiangli S, Cai T, Mburu G, et al. Understanding willingness to use oral pre-exposure prophylaxis for HIV prevention among men who have sex with men in China. Submitted 2016. 10.1371/journal.pone.0199525 PMC6013122 29928008

[46] Xue H, Liu H, Cai L Analysis of willingness and influencing factors for usage of pre-exposure prophylaxis among men who have sex with men. [Chinese] Zhonghua Yu Fang Yi Xue Za Zhi [Chinese Journal of Preventive Medicine]. 2015;49(11):973–77. 26833007

[47] Yang D, Chariyalertsak C, Wongthanee A, Kawichai S, Yotruean K, Saokhieo P, et al Acceptability of pre-exposure prophylaxis among men who have sex with men and transgender women in Northern Thailand. PLoS One. 2013;8(10):e76650. doi:10.1371/journal.pone.0076650 24116132 PMC3792988

[48] Zhang Y, Peng B, She Y, Liang H, Peng H-B, Qian H-Z, et al Attitudes toward HIV pre-exposure prophylaxis among men who have sex with men in Western China. AIDS Patient Care STDS. 2013;27(3):137–41. doi:10.1089/apc.2012.0412 23425017 PMC3595955

[49] Zhou F, Gao L, Li S, Li D, Zhang L, Fan W, et al Willingness to accept HIV pre-exposure prophylaxis among Chinese men who have sex with men. PLoS One. 2012;7(3):e32329. doi:10.1371/journal.pone.0032329 22479320 PMC3316531

[50] Galea J, Kinsler J, Salazar X, Lee SJ, Giron M, Sayles J, et al Acceptability of pre-exposure prophylaxis as an HIV prevention strategy: barriers and facilitators to pre-exposure prophylaxis uptake among at-risk Peruvian populations. Int J STD AIDS. 2011;22(5):256–62. doi:10.1258/ijsa.2009.009255 21571973 PMC3096991

[51] Karuga RN, Njenga SN, Mulwa R, Kilonzo N, Bahati P, O’Reilley K, et al “How I wish this thing was initiated 100 years ago!” willingness to take daily oral pre-exposure prophylaxis among men who have sex with men in Kenya. PLoS One. 2016;11(4):e0151716. doi:10.1371/journal.pone.0151716 27073896 PMC4830617

[52] Chakrapani V, Newman PA, Shunmugam M, Mengle S, Varghese J, Nelson R, et al Acceptability of HIV Pre-Exposure Prophylaxis (PrEP) and implementation challenges among men who have sex with men in India: a qualitative investigation. AIDS Patient Care STDS. 2015 10;29(10):569–77. 26348459 10.1089/apc.2015.0143

[53] Jackson T, Huang A, Chen H, Gao X, Zhong X, Cognitive ZY psychosocial, and sociodemographic predictors of willingness to use HIV pre-exposure prophylaxis among Chinese men who have sex with men. AIDS Behav. 2012 10;16(7):1853–61. doi:10.1007/s10461-012-0188-z 22538373

[54] Validity RG Trustworthiness and rigour: quality and the idea of qualitative research. J Adv Nurs. 2006;53(3):304–10. doi:10.1111/j.1365-2648.2006.03727.x 16441535

[55] Corbin J, Morse JM The unstructured interactive interview: issues of reciprocity and risks when dealing with sensitive topics. Qualit Inquiry. 2003;9(3):335–54.

[56] Saberi P, Gamarel KE, Neilands TB, Comfort M, Sheon N, Darbes LA, et al Ambiguity, ambivalence, and apprehensions of taking HIV-1 pre-exposure prophylaxis among male couples in San Francisco: a mixed methods study. PLoS One. 2012;7(11):e50061. doi:10.1371/journal.pone.0050061 23166819 PMC3498189

[57] Holt M, Murphy DA, Callander D, Ellard J, Rosengarten M, Kippax SC, et al Willingness to use HIV pre-exposure prophylaxis and the likelihood of decreased condom use are both associated with unprotected anal intercourse and the perceived likelihood of becoming HIV positive among Australian gay and bisexual men. Sex Transm Infect. 2012;88(4):258–63. doi:10.1136/sextrans-2011-050312 22290327

[58] Young I, Li J, McDaid L Awareness and willingness to use HIV pre-exposure prophylaxis amongst gay and bisexual men in Scotland: implications for biomedical HIV prevention. PLoS One. 2013;8(5):e64038. doi:10.1371/journal.pone.0064038 23691143 PMC3656929

[59] Frankis J, Young I, Flowers P, McDaid L Who Will Use Pre-Exposure Prophylaxis (PrEP) and Why?: understanding PrEP awareness and acceptability amongst men who have sex with men in the UK–A mixed methods study. PLoS One. 2016;11(4):e0151385. doi:10.1371/journal.pone.0151385 27093430 PMC4836740

[60] Rocha LM, Campos MJ, Brito J, Fuertes R, Rojas J, Pinto N, et al Acceptability of PrEP among HIV negative Portuguese men who have sex with men that attended 2014 Lisbon pride fair. J Int AIDS Soc. 2014;17(4 Suppl 3):19734. doi:10.7448/IAS.17.4.19734 25397480 PMC4225295

[61] Ferrer L, Folch C, Fernandez-Davila P, Garcia A, Morales A, Belda J, et al Awareness of pre-exposure prophylaxis for HIV, willingness to use it and potential barriers or facilitators to uptake among men who have sex with men in Spain. AIDS Behav. 2016;20(7):1423–33. doi:10.1007/s10461-016-1379-9 27022938

[62] Kesler MA, Kaul R, Myers T, Liu J, Loutfy M, Remis RS, et al Perceived HIV risk, actual sexual HIV risk and willingness to take pre-exposure prophylaxis among men who have sex with men in Toronto, Canada. AIDS Care. 2016;1–8. doi:10.1080/09540121.2016.1178703 27136725

[63] Lebouche B, Engler K, Machouf N, Lessard D, Thomas R Predictors of interest in taking pre-exposure prophylaxis among men who have sex with men who used a rapid HIV-testing site in Montreal (Actuel sur Rue). HIV Med. 2016;17(2):152–58. doi:10.1111/hiv.12286 26177691

[64] Grov C, Whitfield TH, Rendina HJ, Ventuneac A, Parsons JT Willingness to take PrEP and potential for risk compensation among highly sexually active gay and bisexual men. AIDS Behav. 2015;19(12):2234–44. doi:10.1007/s10461-015-1030-1 25735243 PMC4560674

[65] Goedel WC, Halkitis PN, Greene RE, Duncan DT Correlates of awareness of and Willingness to Use Pre-exposure Prophylaxis (PrEP) in gay, bisexual, and other men who have sex with men who use geosocial-networking smartphone applications in New York City. AIDS Behav. 2016;20(7):1435–42. doi:10.1007/s10461-016-1353-6 26966013

[66] Mitchell JW, Stephenson R HIV-negative partnered men’s willingness to use pre-exposure prophylaxis and associated factors among an internet sample of U.S. HIV-negative and HIV-discordant male couples. LGBT Health. 2015;2(1):35–40. doi:10.1089/lgbt.2014.0092 26790016 PMC4855775

[67] Crosby RA, Geter A, DiClemente RJ, Salazar LF Acceptability of condoms, circumcision and PrEP among young black men who have sex with men: a descriptive study based on effectiveness and cost. Vaccines. 2014;2(1):129–37. doi:10.3390/vaccines2010129 26344471 PMC4494197

[68] Hoots BE, Finlayson T, Nerlander L, Paz-Bailey G Willingness to take, use of, and indications for pre-exposure prophylaxis among men who have sex with men-20 US cities, 2014. Clin Infect Dis. 2016. doi:10.1093/cid/ciw367 PMC5009903 27282710

[69] Fallon SA, Park JN, Ogbue CP, Flynn C, German DA Awareness and acceptability of pre-exposure HIV prophylaxis among men who have sex with men in Baltimore. AIDS Behav. 2016. doi:10.1007/s10461-016-1619-z PMC13211940 27873081

[70] Cohen SE, Vittinghoff E, Bacon O, Doblecki-Lewis S, Postle BS, Feaster DJ, et al High interest in preexposure prophylaxis among men who have sex with men at risk for HIV infection: baseline data from the US PrEP demonstration project. J Acquir Immune Defic Syndr. 2015;68(4):439–48. doi:10.1097/QAI.0000000000000479 25501614 PMC4334721

[71] Glidden DV, Buchbinder SP, Anderson PL, McMahan V, Amico KR, Liu A, et al PrEP engagement for HIV prevention: results from the iPrEx open label extension (OLE). Top Antivir Med. 2015;23:445.

[72] Hosek S, Martinez J, Santos K, Mehrotra M, Balthazar C, Serrano P, et al PrEP interest, uptake, and adherence among young men who have sex with men (YMSM) in the USA. Top Antivir Med. 2014;22(e–1):498.

[73] Krakower DS, Mimiaga MJ, Rosenberger JG, Novak DS, Mitty JA, White JM, et al Limited awareness and low immediate uptake of pre-exposure prophylaxis among men who have sex with men using an internet social networking site. PLoS One. 2012;7(3):e33119. doi:10.1371/journal.pone.0033119 22470438 PMC3314648

[74] Kojima N, Davey DJ, Klausner JD Pre-exposure prophylaxis for HIV infection and new sexually transmitted infections among men who have sex with men. Aids. 2016;30(14):2251–52. doi:10.1097/qad.0000000000001185 27314179

[75] Sagaon-Teyssier L, Suzan-Monti M, Demoulin B, Capitant C, Lorente N, Preau M, et al Uptake of PrEP and condom and sexual risk behavior among MSM during the ANRS IPERGAY trial. AIDS Care. 2016;28(Suppl 1):48–55. doi:10.1080/09540121.2016.1146653 26883400 PMC4828609

[76] Wilton J, Kain T, Fowler S, Hart TA, Grennan T, Maxwell J, et al Use of an HIV-risk screening tool to identify optimal candidates for PrEP scale-up among men who have sex with men in Toronto, Canada: disconnect between objective and subjective HIV risk. J Int AIDS Soc. 2016;19(1):20777. doi:10.7448/ias.19.1.20777 27265490 PMC4911732

[77] Greene GJ, Swann G, Fought AJ, Carballo-Dieguez A, Hope TJ, Kiser PF, et al Preferences for Long-Acting Pre-exposure Prophylaxis (PrEP), daily oral PrEP, or condoms for HIV prevention among U.S. men who have sex with men. AIDS Behav. 2016. doi:10.1007/s10461-016-1565-9 PMC5380480 27770215

[78] Holtz TH, Chitwarakorn A, Curlin ME, Hughes J, Amico KR, Hendrix C, et al HPTN 067/ADAPT study: a comparison of daily and non-daily pre-exposure prophylaxis dosing in Thai men who have sex with men, Bangkok, Thailand. J Int AIDS Soc. 2015;18:25–26. doi:10.7448/IAS.18.5.20539

[79] Hosek SG, Siberry G, Bell M, Lally M, Kapogiannis B, Green K, et al The acceptability and feasibility of an HIV preexposure prophylaxis (PrEP) trial with young men who have sex with men. J Acquir Immune Defic Syndr. 2013 4 1;62(4):447–56. doi:10.1097/QAI.0b013e3182801081 24135734 PMC3656981

[80] Haire BG Preexposure prophylaxis-related stigma: strategies to improve uptake and adherence - a narrative review. Hiv Aids. 2015;7:241–49. doi:10.2147/HIV.S72419 PMC4610795 26508889

[81] Krakower DS, Mayer KH Pre-exposure prophylaxis to prevent HIV infection: current status, future opportunities and challenges. Drugs. 2015;75(3):243–51. doi:10.1007/s40265-015-0355-4 25673022 PMC4354703

[82] Mburu G, Ram M, Skovdal M, Bitira D, Hodgson I, Mwai G, et al Resisting and challenging stigma in Uganda: the role of support groups of people living with HIV. J Int AIDS Soc. 2013;16(3 Suppl 2):18636. doi:10.7448/IAS.16.3.18636 24242256 PMC3833188

[83] Ross I, Mejia C, Melendez J, Chan PA, Nunn AC, Powderly W, et al Awareness and attitudes of pre-exposure prophylaxis for HIV prevention among physicians in Guatemala: implications for country-wide implementation. PLoS One. 2017;12(3):e0173057. doi:10.1371/journal.pone.0173057 28257475 PMC5336255

[84] van der Elst EM, Gichuru E, Muraguri N, Musyoki H, Micheni M, Kombo B, et al Strengthening healthcare providers’ skills to improve HIV services for MSM in Kenya. Aids. 2015;29(Suppl 3):S237–40. doi:10.1097/qad.0000000000000882 26372492 PMC4706371

[85] Caceres CF, Borquez A, Klausner JD, Baggaley R, Beyrer C Implementation of pre-exposure prophylaxis for human immunodeficiency virus infection: progress and emerging issues in research and policy. J Int AIDS Soc. 2016;19(7(Suppl6)):21108. doi:10.7448/ias.19.7.21108 27760685 PMC5071779

[86] Chan PA, Mena L, Patel R, Oldenburg CE, Beauchamps L, Perez-Brumer AG, et al Retention in care outcomes for HIV pre-exposure prophylaxis implementation programmes among men who have sex with men in three US cities. J Int AIDS Soc. 2016;19:8. doi:10.7448/ias.19.1.20903 PMC4908080 27302837

[87] King HL, Keller SB, Giancola MA, Rodriguez DA, Chau JJ, Young JA, et al Pre-exposure prophylaxis accessibility research and evaluation (PrEPARE Study). AIDS Behav. 2014;18(9):1722–25. doi:10.1007/s10461-014-0845-5 25017425 PMC4127131

[88] Holloway IW, Dougherty R, Gildner J, Beougher SC, Pulsipher C, Montoya JA, et al Brief report: PrEP uptake, adherence, and discontinuation among California YMSM using geosocial networking applications. J Acquir Immune Defic Syndr. 2017;74(1):15–20. doi:10.1097/qai.0000000000001164 27552158 PMC5140696

[89] Brooks RA, Landovitz RJ, Regan R, Lee SJ, Allen VC Perceptions of and intentions to adopt HIV pre-exposure prophylaxis among black men who have sex with men in Los Angeles. Int J STD AIDS. 2015;26(14):1040–48. doi:10.1177/0956462415570159 25638214 PMC4520772

[90] Krumpal I Determinants of social desirability bias in sensitive surveys: a literature review. Qual Quant. 2013;47(4):2025–47.

[91] Mugo NR, Ngure K, Kiragu M, Irungu E, Kilonzo N The preexposure prophylaxis revolution; from clinical trials to programmatic implementation. Curr Opin HIV AIDS. 2016;11(1):80–86. doi:10.1097/COH.0000000000000224 26575147 PMC4900687

[92] Ravasi G, Grinsztejn B, Baruch R, Guanira JV, Luque R, Caceres CF, et al Towards a fair consideration of PrEP as part of combination HIV prevention in Latin America. J Int AIDS Soc. 2016;19(Suppl 6):21113. doi:10.7448/ias.19.7.21113 27760687 PMC5071748

[93] Beyrer C, Baral SD, Collins C, Richardson ET, Sullivan PS, Sanchez J, et al The global response to HIV in men who have sex with men. Lancet. 2016;388(10040):198–206. doi:10.1016/S0140-6736(16)30781-4 27411880

